# Hesperetin Rescues Amyloid Beta-Induced Defects in Neurite Outgrowth Under *In Vitro* Mild Cognitive Impairment-like Cellular Conditions

**DOI:** 10.3390/ijms27125481

**Published:** 2026-06-17

**Authors:** Asahi Honjo, Hideji Yako, Mizuki Kasai, Mikako Chiba, Ayano Satsuka, Tomohisa Kato, Moeri Yagi, Akinori Nishi, Yuki Miyamoto, Junji Yamauchi

**Affiliations:** 1 Laboratory of Molecular Neuroscience and Neurology, Tokyo University of Pharmacy and Life Sciences, Hachioji, Tokyo 192-0392, Japanhyako@toyaku.ac.jp (H.Y.);; 2 Strategic Planning Department, Oji Holdings Corporation, Koto, Tokyo 135-8558, Japan; 3 Tsumura Advanced Technology Research Laboratories, Tsumura & Co., Inashiki, Ibaraki 200-1192, Japan; 4 Department of Pharmacology, National Research Institute for Child Health and Development, Setagaya, Tokyo 157-8535, Japan; 5 Department of Biological Science, Tokyo College of Biotechnology, Oota, Tokyo 144-0032, Japan

**Keywords:** amyloid beta, Alzheimer’s disease, in vitro, hesperetin, neuronal cell, differentiation

## Abstract

Accumulation of aggregated amyloid beta (Aβ) species is a defining pathological hallmark of Alzheimer’s disease and is associated with extensive neuronal structural abnormalities. Mild cognitive impairment (MCI), a transitional stage between normal aging and the onset of dementia, is thought to represent an early phase of this pathological continuum. Studies at the cellular level suggest that the conditions impair the maintenance of established neuronal processes/networks and restrict their capacity for elongation or re-elongation. They may also attenuate the activation and process extension of quiescent neural progenitor or stem-like cells. These early cellular changes precede overt neurodegeneration in neural tissue and are likely to contribute to cognitive decline. They highlight the importance of in vitro models for identifying molecular targets involved in recovery from disease. In this study, we investigated the effects of aggregated Aβ (25–35) on neuronal process elongation and associated intracellular events in the N1E-115 cell line, a widely used model of neuronal differentiation. Addition of aggregated Aβ to cultured N1E-115 cells attenuated process elongation in a concentration-dependent manner. This morphological impairment was accompanied by decreased expression of neuronal differentiation markers. In contrast, at the half-maximal inhibitory concentration for process elongation, long-term cultured cells did not exhibit apparent process retraction or degenerative morphology. This mild but progressive impairment, without extensive cell death, is consistent with the cellular features of early-stage conditions rather than advanced Alzheimer’s pathologies. Similar results were observed in primary cortical neurons. Aβ also decreased the level of GTP-bound Ras and phosphorylation of the downstream mitogen-activated protein kinase/extracellular signal-regulated kinase (MAPK/ERK). Furthermore, treatment with hesperetin, a bioactive flavonoid compound, recovered the Aβ-induced inhibition of neuronal process elongation. Hesperetin also restored Ras and MAPK/ERK states, suggesting that its effects are associated, at least in part, with modulation of signaling through Ras and MAPK/ERK. Our findings suggest that hesperetin may serve as a useful molecular probe for modulating early cellular responses associated with Alzheimer’s disease-related pathology. This in vitro model might serve as a useful platform for investigating the molecular target candidates involved in recovery from nervous system disorders.

## 1. Introduction

Alzheimer’s disease (AD) is the most common cause of dementia worldwide and is characterized pathologically by the accumulation of aggregated amyloid beta (Aβ) species in the brain. These aggregates are not only hallmarks of the disease but also active drivers of neuronal dysfunction [1,2,3,4]. Aggregated Aβ accumulation disrupts neuronal networks and synaptic integrity, leading to progressive alterations in neuronal morphologies and brain tissue architectures [1,2,3,4]. It also impairs intracellular signaling pathways that are essential for neuronal cell morphogenesis and plasticity, as well as cell survival [1,2,3,4]. Importantly, such cellular disturbances arise long before extensive neuronal loss becomes apparent, indicating that early-stage molecular and structural abnormalities play a critical role in the initiation and progression of AD [3,4,5,6].

Prior to the development of overt dementia, during which mid-sized to large aggregates of Aβ accumulate in brain regions such as the frontal lobe, the brain experiences a prolonged phase of subtle yet progressive cellular alterations [7,8,9]. During this phase, referred to as mild cognitive impairment (MCI), neuronal cells exhibit reduced neuronal process elongation/network formation and compromised synaptic connectivity, often accompanied by inflammatory dysregulation in certain glial populations [7,8,9]. In addition, the intrinsic capacity for axonal elongation in mature neuronal cells or quiescent neural progenitor/stem-like cells may be progressively attenuated at this stage. It limits the ability of neural circuits to compensate for early structural and functional deficits in some brain regions. These early alterations reduce neuronal connectivity and may impair circuit-level compensation, ultimately weakening overall network integrity and contributing to the gradual decline in cognitive function [7,8,9,10,11,12]. Some of these events occur at a stage when neuronal cells remain largely viable. Therefore, this period may represent a critical window of opportunity for investigating molecular mechanisms underlying restoration of cellular and circuit function rather than merely preventing cell death [7,8,9,10,11,12].

Neurite outgrowth is a key determinant of neuronal connectivity and information processing in the central nervous system (CNS) [13,14,15,16,17,18]. Its disruption is directly linked to compromised synaptic communication and circuit formation [5,6,19,20]. Among the signaling pathways that regulate neuronal process elongation, the Ras and mitogen-activated protein kinase/extracellular signal-regulated kinase (MAPK/ERK) cascade represents a central pathway involved in neuronal differentiation and process elongation [21,22]. Impairment of Ras and MAPK/ERK signaling has been associated with disturbances in neuronal differentiation, neurite outgrowth, and neuronal maintenance, which may contribute to neurodegenerative processes [23,24]. Since these pathways play central roles in regulating neuronal structure and function as well as neuronal survival [21,22,23,24], understanding how they are affected by Aβ aggregation is of considerable interest. However, the mechanisms through which Aβ aggregates influence this signaling axis during the early stages of neuronal dysfunction remain to be fully elucidated.

In parallel with mechanistic studies, increasing attention has been directed toward small bioactive molecules with the potential to modulate early AD-related cellular abnormalities [25,26]. Naturally derived flavonoids, including hesperetin (also called vitamin P aglycon), have attracted particular attention because of their biological activities, including modulation of oxidative stress and intracellular signaling pathways [25,26,27,28,29,30]. Hesperetin is reported to modulate intracellular signaling, oxidative stress, and inflammation in the CNS [25,26,27,28,29,30]. Traditional studies have primarily focused on the descriptive efficacy of flavonoids. However, recent advances in biomedical applications have expanded their therapeutic potential. Advanced drug delivery systems, including polymeric hydrogels, nano-formulations, and nano-vesicles, are being developed to overcome the poor solubility and limited blood–brain barrier permeability of lipophilic polyphenols, thereby enhancing targeted neuroprotective delivery [27,28]. Nevertheless, although hesperetin has been reported to influence cellular responses to various neurotoxic insults [25,26,27,28,29,30], its ability to restore aggregated Aβ-induced impairments in neuronal process elongation and the associated signaling pathways has not been examined in a model of early effects of aggregated Aβ on neuronal cell differentiation.

Here, we address these issues using primary cortical neurons [31,32] and the N1E-115 cell line, a neuroblastoma-derived cell line exhibiting progenitor-like neuronal differentiation properties [33,34,35,36]. We have investigated how the aggregated Aβ core amino acid sequence [37,38,39,40] affects cell morphogenesis as well as Ras and MAPK/ERK signaling, and whether hesperetin is able to reverse these early pathological changes in these cells. By focusing on molecular and structural abnormalities that precede overt neurodegeneration, we highlight a potentially reversible stage of possibly early AD-related cellular alterations. Our findings suggest that hesperetin modulates early neuronal alterations induced by aggregated Aβ, and that this in vitro system provides a valuable platform for exploring potential molecular targets involved in recovery from neurodegenerative disorders.

## 2. Results

### 2.1. Aggregated Aβ (25–35) Species Inhibit Process Elongation in Cells Following the Induction of Differentiation

**Figure 1:**
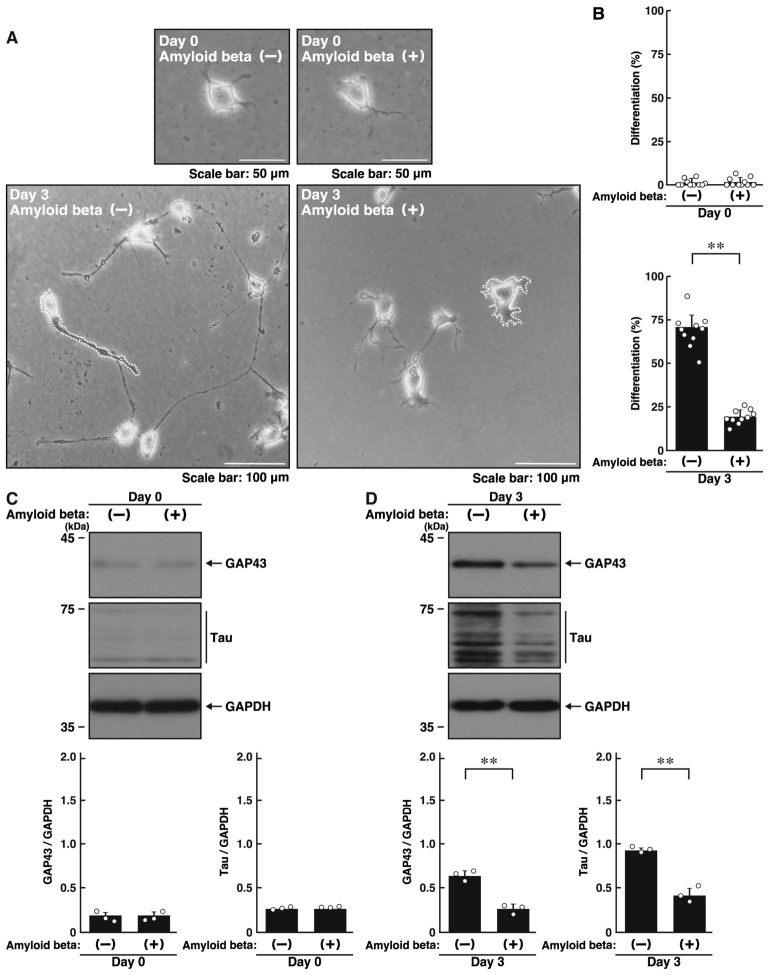
Addition of aggregated Aβ (25–35) inhibits morphological differentiation in N1E-115 cells. (**A**,**B**) N1E-115 cells were treated with (+) aggregated Aβ (25–35) or vehicle control (−) at half-maximal inhibitory concentration (13 µM). Cells were allowed to differentiate morphologically for 0 or 3 days (white dotted lines surround typical morphologically differentiated or undifferentiated cells). Cells with processes were counted as differentiated and depicted in the graph (** *p* < 0.01; n = 10 fields). (**C**,**D**) Following the induction of differentiation, cells were collected at days 0 and 3, lysed, and immunoblotted with antibodies against GAP43, Tau, and an internal control protein (GAPDH). The quantified immunoreactive bands were normalized to internal control proteins (** *p* < 0.01; n = 3).

First, we investigated the effects of aggregated Aβ (25–35) species on process elongation during differentiation of the N1E-115 cell line, a neuroblastoma-derived line exhibiting progenitor-like neuronal differentiation properties, under the short-term cultured conditions (Figure 1A). The N1E-115 cell line is a well-established model for studying the molecular mechanisms of neurite outgrowth and signaling pathways during neuronal maturation [31,32]. The experimental model was intended to mimic a condition in which differentiating cells are exposed to aggregated Aβ during ongoing morphological maturation. It has been suggested that MCI-like conditions can compromise the maintenance of established neuronal networks composed of elongated processes, limit their ability to elongate or re-elongate, and may further suppress the activation and process extension of quiescent neural progenitor or stem-like cells [7,10]. To simulate the neuronal impairment, we employed Aβ (25–35), the shortest fragment retaining full neurotoxicity and characterized by its high propensity for rapid aggregation [37,38].

**Figure 2:**
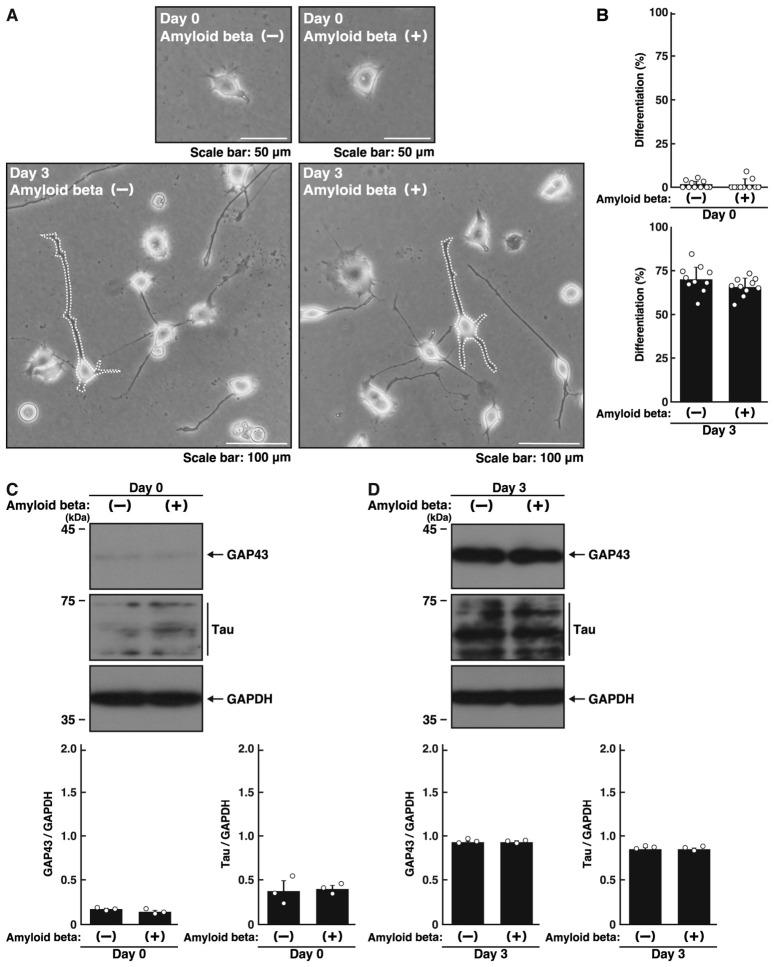
Addition of soluble Aβ (25–35) does not significantly change morphological differentiation in N1E-115 cells. (**A**,**B**) N1E-115 cells were treated with (+) 13 µM soluble Aβ (25–35) or vehicle control (−). Cells were allowed to differentiate morphologically for 0 or 3 days (white dotted lines surround typical morphologically differentiated cells). Cells with processes were counted as differentiated and depicted in the graph (n = 10 fields). (**C**,**D**) Following the induction of differentiation, cells were collected at days 0 and 3, lysed, and immunoblotted with antibodies against GAP43, Tau, and GAPDH. The quantified immunoreactive bands were normalized to internal control proteins (n = 3).

Aggregated Aβ inhibited process elongation and increased the number of trypan blue-positive cells (i.e., dying cells) in a concentration-dependent manner (Figure 1B; Figure S1). In this short-term culture condition, half-maximal inhibitory concentration was approximately 13 µM. Decreased expression of the neuronal differentiation markers growth-associated protein 43 (GAP43) and Tau, both of which are critical for microtubule stabilization and axonal growth [15,16], was observed following treatment with aggregated Aβ (Figure 1C,D). We also checked the expression levels of synaptic markers synaptophysin and postsynaptic density protein 95 (PSD95). Decreased expression levels of these proteins were also observed following treatment with aggregated Aβ (Figure S2). Similarly, treatment with aggregated full-length Aβ (1–42) species also decreased the expression levels of synaptophysin and postsynaptic PSD95 (Figure S3). In contrast, treatment with soluble Aβ (25–35) did not produce any apparent changes in cell morphology following the induction of differentiation (Figure 2A,B). Consistently, the expression levels of GAP43 and Tau were not significantly altered by soluble Aβ (25–35) treatment (Figure 2C,D).

**Figure 3:**
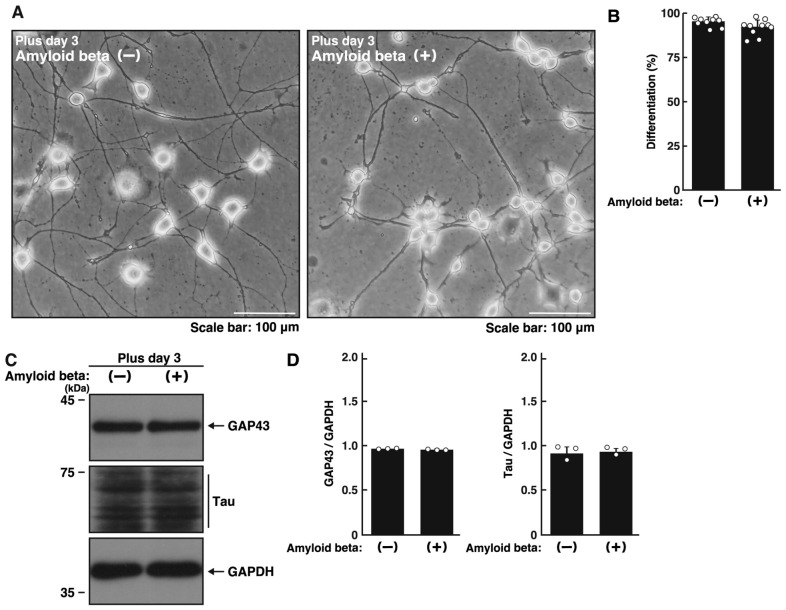
Aggregated Aβ does not significantly affect morphological differentiation in long-term cultured N1E-115 cells. (**A**,**B**) N1E-115 cells were allowed to differentiate to promote the extension of long processes. Then, cells were treated without (−, vehicle control) or with (+) 13 µM aggregated Aβ (25–35). Cells were allowed to differentiate morphologically for an additional 3 days. Cells with processes were counted as differentiated and depicted in the graph (n = 10 fields). (**C**,**D**) Cells were collected, lysed, and immunoblotted with antibodies against GAP43, Tau, and GAPDH. The quantified immunoreactive bands were normalized to internal control proteins (n = 3).

**Figure 4:**
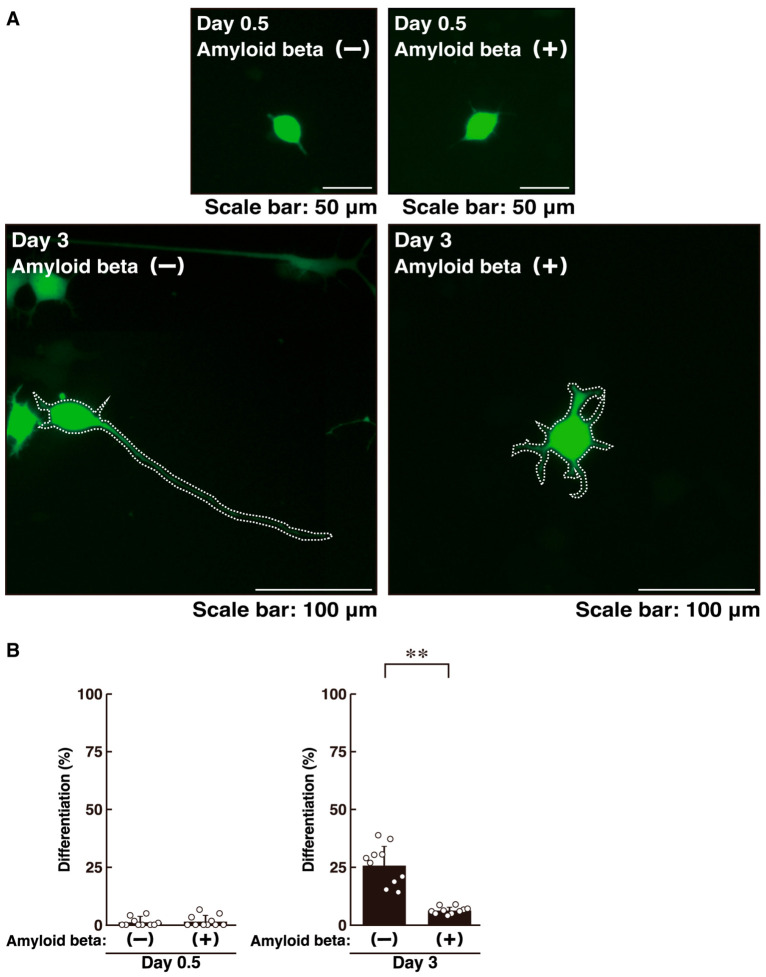
Addition of aggregated Aβ inhibits neurite elongation in primary cortical neurons in short-term culture. (**A**,**B**) Primary cortical neurons, which were transfected with the plasmid encoding enhanced GFP to identify cell morphologies, were treated with (+) or without (−, vehicle control) aggregated Aβ (25–35) at 13 µM. Neuron culture continued for 0.5 or 3 days. Typical differentiated or undifferentiated cells are surrounded by dotted white lines. Cells with differentiated phenotypes are depicted in the graph (** *p* < 0.01; n = 10 fields).

**Figure 5:**
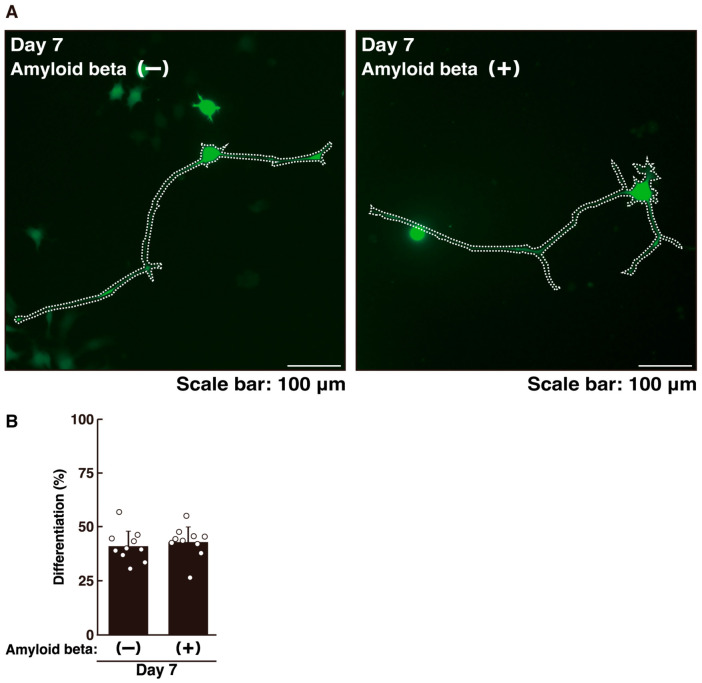
The effect of aggregated Aβ on primary cortical neurons in the long-term culture condition. (**A**,**B**) Primary cortical neurons transfected with the plasmid encoding enhanced GFP to identify cell morphologies were cultured for 1 week. Then, neurons were treated with or without 13 µM aggregated Aβ (25–35) for 3 days. Typical differentiated cells are surrounded by dotted white lines. Cells with differentiated phenotypes are depicted in the graph (n = 10 fields).

Treatment of long-term cultures with aggregated Aβ at 13 µM did not result in any apparent morphological changes or a decrease in marker expression (Figure 3A–D). This experiment was designed to model a condition in which even low levels of aggregated Aβ are present in neuronal cells that have already formed mature processes, possibly compromising their intrinsic capacity for additional process elongation rather than causing marked degeneration of pre-existing neurites. Together, these findings indicate progressive morphological decline in the absence of extensive neuronal loss, which is consistent with the cellular features observed in early AD pathology rather than those in advanced pathology [1,7]. Also, under both the short-term (Figure 4A,B) and long-term (Figure 5A,B) culture conditions, the results observed in N1E-115 cells were largely reproduced in primary cortical neurons, further validating the physiological relevance of our observations in neuronal models [33,34].

**Figure 6:**
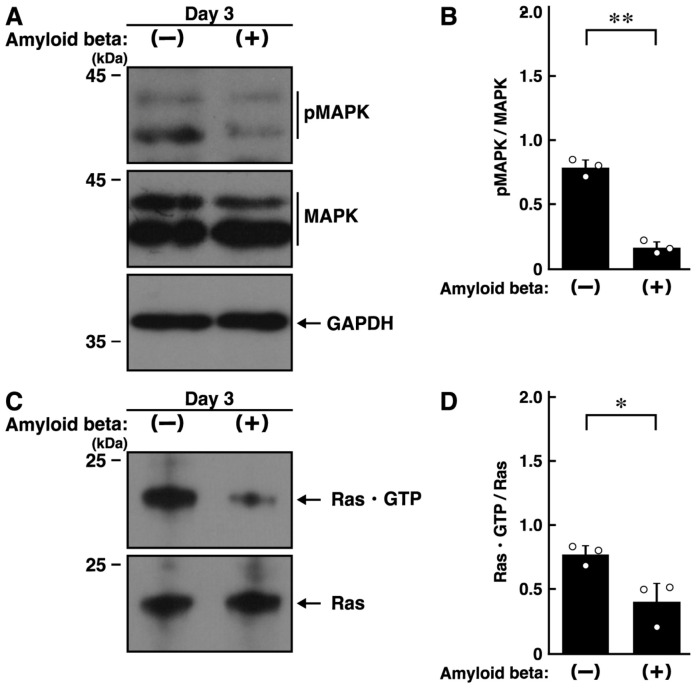
Addition of aggregated Aβ decreases the phosphorylation of MAPK/ERK and levels of the GTP-bound form of Ras. (**A**,**B**) N1E-115 cells were allowed to differentiate in the presence (+) or absence (−, vehicle control) of aggregated Aβ (13 µM) for 3 days. Cells were then collected and lysed for immunoblotting using antibodies against phosphorylated MAPK/ERK (pMAPK), MAPK/ERK (MAPK), or GAPDH. The quantified immunoreactive bands were normalized to control total proteins (** *p* < 0.01; n = 3). (**C**,**D**) Cell lysates were used for the pull-down assay to detect active, GTP-bound Ras. Total Ras was also detected in immunoblotting (GTP-bound Ras was normalized to total Ras; * *p* < 0.05; n = 3).

We next examined whether, following induction of differentiation in N1E-115 cells, aggregated Aβ affects MAPK/ERK, one of the major regulators of neuronal differentiation and neurite initiation [23,24]. Addition of Aβ to cells was associated with decreased MAPK/ERK phosphorylation (Figure 6A,B). Similarly, addition of Aβ led to a decrease in the active, GTP-bound form of Ras molecular species (N-Ras, H-Ras, and K-Ras) (Figure 6C,D), the major upstream regulators of MAPK/ERK involved in translating extracellular cues into morphological changes [41,42,43].

Collectively, these results suggest that aggregated Aβ species-induced impairment of neuronal morphological differentiation is observed and is mediated, at least in part, through the MAPK/ERK signaling pathway.

### 2.2. Treatment with Hesperetin Recovers Aggregated Aβ-Induced Inhibition of Process Elongation

**Figure 7:**
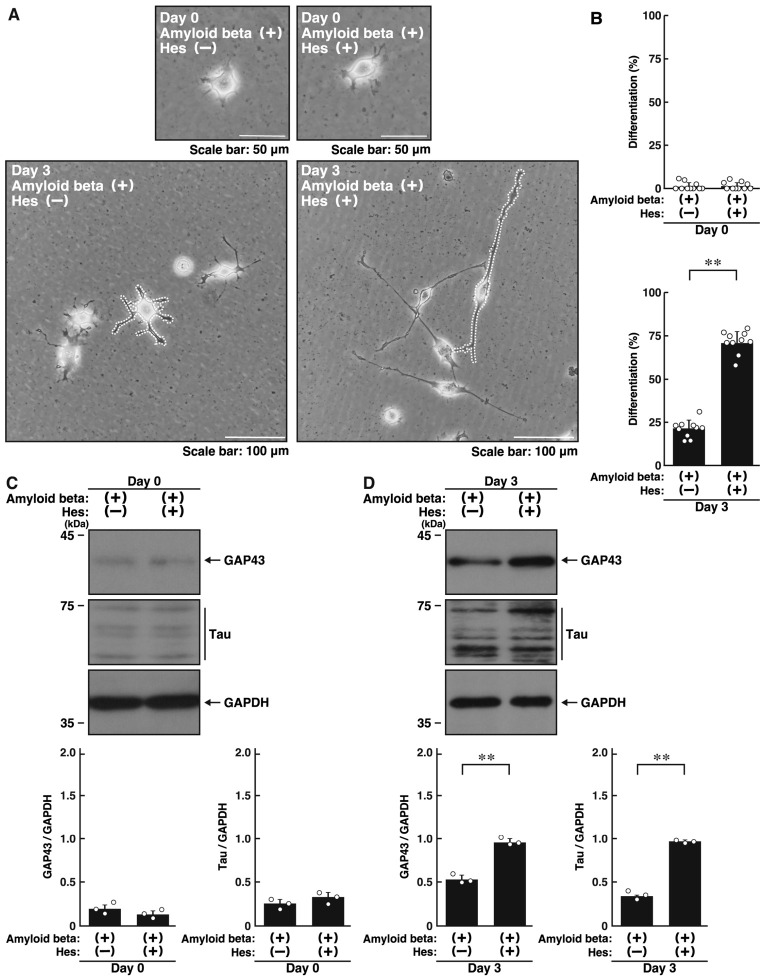
Hesperetin recovers the effects of aggregated Aβ on morphological differentiation. (**A**,**B**) N1E-115 cells were treated with aggregated Aβ at half-maximal inhibitory concentration (13 µM) in the presence (+) or absence (−, vehicle control) of 10 µM hesperetin (Hes). Cells were allowed to differentiate morphologically for 0 or 3 days (white dotted lines surround typical morphologically differentiated or undifferentiated cells). Cells with processes were counted as differentiated and depicted in the graph (** *p* < 0.01; n = 10 fields). (**C**,**D**) Following the induction of differentiation, cells were collected at days 0 and 3, lysed, and immunoblotted with antibodies against GAP43, Tau, and GAPDH. The quantified immunoreactive bands were normalized to internal control proteins (** *p* < 0.01; n = 3).

**Figure 8:**
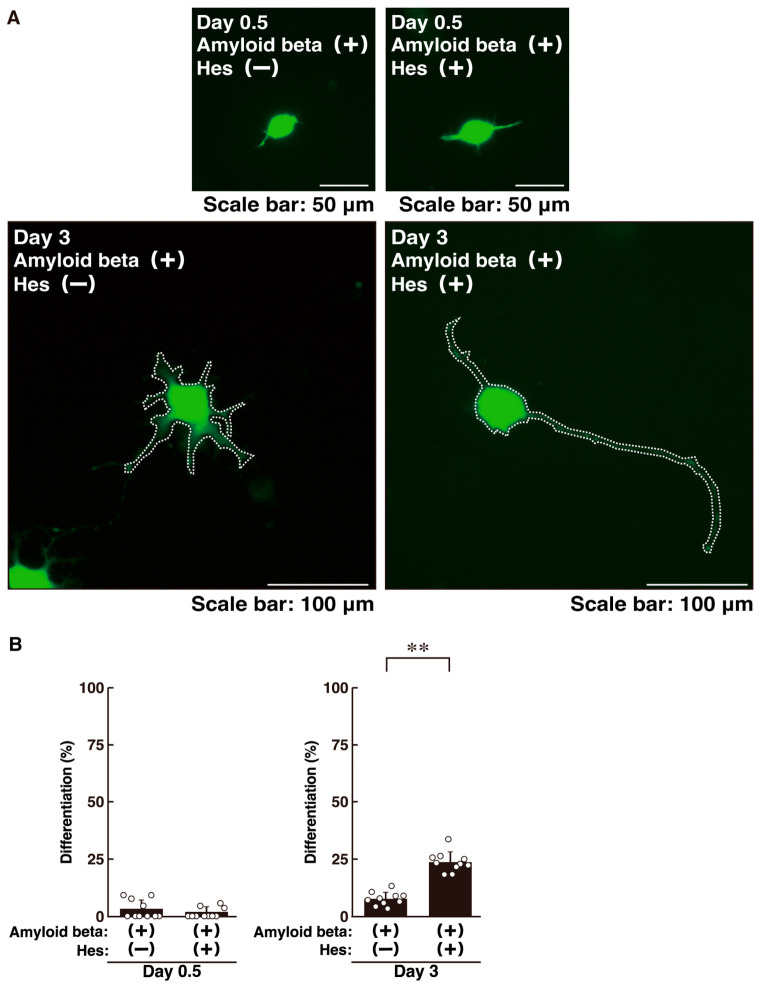
Hesperetin recovers aggregated Aβ-induced inhibition of neurite elongation in primary cortical neurons. (**A**,**B**) Primary cortical neurons, which were transfected with the plasmid encoding enhanced GFP to identify cell morphologies, were treated with 13 µM aggregated Aβ (25–35) in the presence (+) or absence (−, vehicle control) of 10 µM hesperetin (Hes). Neuron culture continued for 0.5 or 3 days. Typical differentiated or undifferentiated cells are surrounded by dotted white lines. Cells with differentiated phenotypes are depicted in the graph (** *p* < 0.01; n = 10 fields).

Next, we examined whether hesperetin could counteract the inhibitory effects of aggregated Aβ on cells following the induction of differentiation. Hesperetin and its derivatives have been reported to exhibit neuroprotective properties and modulate intracellular signaling pathways in various neurodegenerative contexts [25,26,27,28,29,30]. Because aggregated Aβ did not significantly affect long-term cultured cells in previous experiments, we did not examine long-term cultures in the following set of experiments. In differentiating cells, co-treatment with hesperetin markedly restored Aβ-induced suppression of process elongation (Figure 7A,B). Similar results were observed in primary cortical neurons (Figure 8A,B).

Hesperetin ameliorated the aggregated Aβ-induced suppression of process elongation in a concentration-dependent manner, and a significant restorative effect was observed at 10 μM (Figure S4). Moreover, release of cytochrome c was minimal at this concentration, indicating that it is suitable for further analyses. Therefore, hesperetin was used at 10 μM in the subsequent experiments. The Aβ-induced reductions in GAP43 and Tau, as well as synaptophysin and PSD95 levels, were substantially reversed by hesperetin (Figure 7C,D; Figure S2). To further assess the involvement of oxidative stress, we examined the expression of heme oxygenase 1 (HO-1), an oxidative stress-responsive cytoprotective protein, and the level of 4-hydroxy-2-nonenal (4-HNE) modification [25,26]. Aggregated Aβ increased both HO-1 expression and 4-HNE modification [25,26], suggesting enhanced oxidative stress, whereas hesperetin treatment markedly attenuated these changes (Figure S2). These results provide additional evidence that hesperetin mitigates Aβ-induced oxidative stress, supporting the notion that the suppression of oxidative stress by flavonoids often contributes to their neuroprotective effects [25,26].

N1E-115 cells cultured on CellArray-Heart dishes exhibited a highly aligned morphology, allowing clear discrimination between cell bodies with short protrusions and elongated neurite-like processes in microscopic images (Figure S5). The micropatterned surface containing alternating conventional and nanostructured regions promoted preferential localization of cell bodies to nanostructured regions, resulting in highly aligned cell morphology. This culture system enabled robust quantification of process extension across a groove without fixation. Aggregated Aβ markedly impaired process elongation, whereas hesperetin recovered this defect. Importantly, the combination of the aligned culture substrate and image-based analysis provided a simple and efficient method for separately evaluating cell body and neurite-like morphology, thereby facilitating quantitative assessment of Aβ-induced neurite impairment and its recovery by hesperetin in vitro.

**Figure 9:**
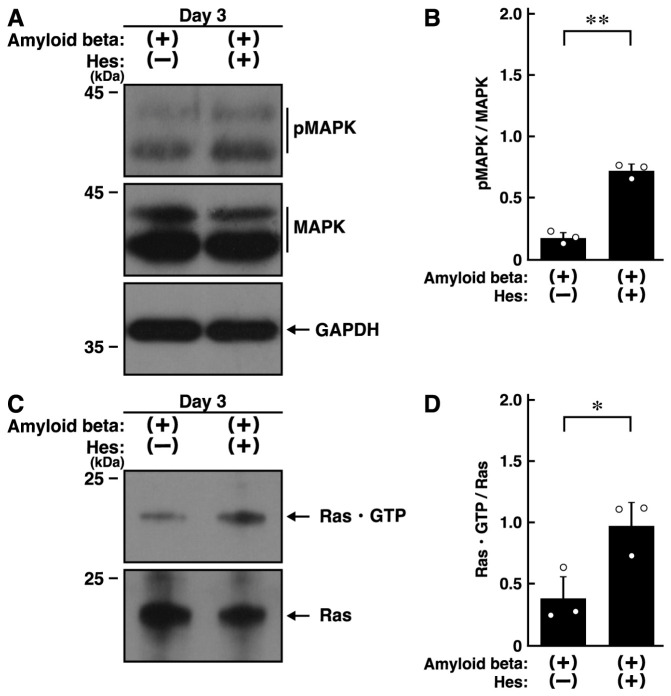
Hesperetin recovers the effects of aggregated Aβ on MAPK phosphorylation and levels of GTP-bound Ras. (**A**,**B**) Aggregated Aβ (13 µM)-treated N1E-115 cells were allowed to differentiate in the presence (+) or absence (−, vehicle control) of hesperetin (Hes, 10 µM) for 3 days. Cells were then collected and lysed for immunoblotting using antibodies against phosphorylated MAPK/ERK (pMAPK), MAPK/ERK (MAPK), or GAPDH. The quantified immunoreactive bands were normalized to control total proteins (** *p* < 0.01; n = 3). (**C**,**D**) Cell lysates were used for the pull-down assay to detect active, GTP-bound Ras. Total Ras was also detected in immunoblotting (GTP-bound Ras was normalized to total Ras; * *p* < 0.05; n = 3).

**Figure 10:**
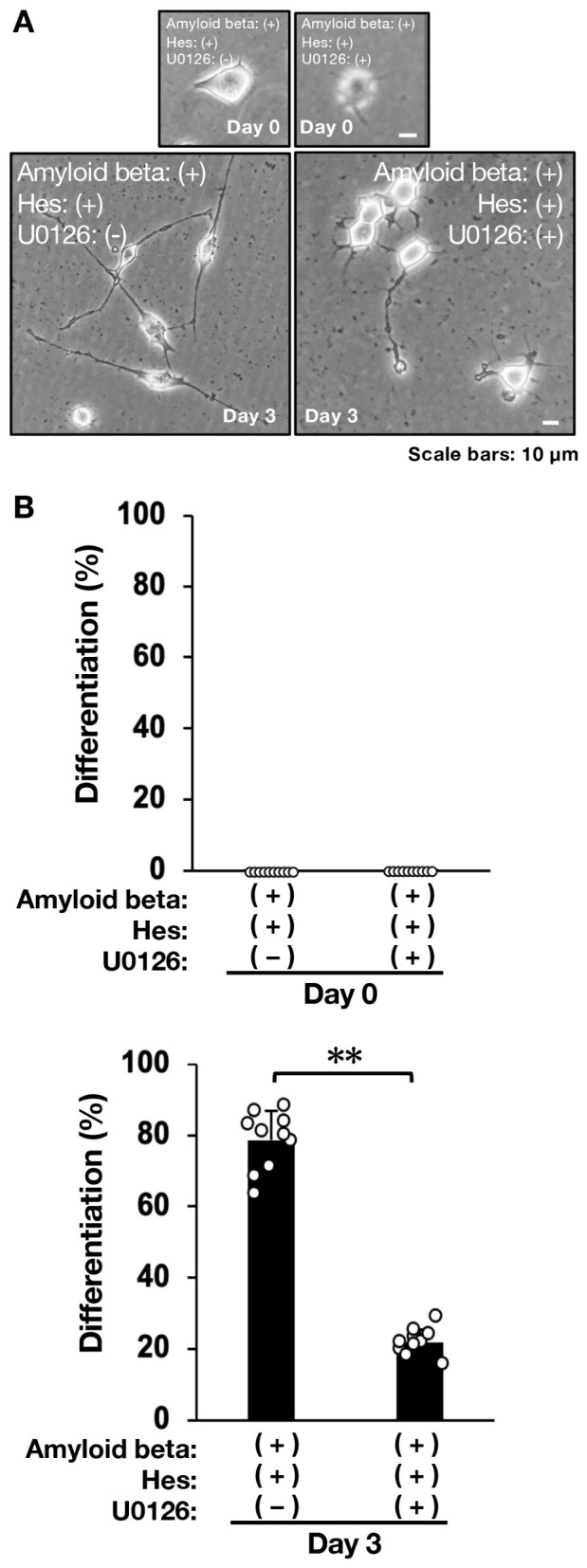
The effect of hesperetin is mediated, at least in part, by MAPK/ERK signaling. (**A**,**B**) N1E-115 cells were treated with aggregated Aβ at half-maximal inhibitory concentration (13 µM) in the presence of 10 µM hesperetin (Hes) with (+) or without (−,vehicle control) of U0126, an inhibitor of MEK1/2. Cells were allowed to differentiate morphologically for 0 or 3 days. Cells with processes were counted as differentiated and depicted in the graph (** *p* < 0.01; n = 10 fields).

Furthermore, hesperetin restored the decrease in both MAPK/ERK phosphorylation (Figure 9A,B) and the levels of GTP-bound Ras (Figure 9C,D), which were caused by Aβ. Additionally, we asked whether the effect of hesperetin depends on signaling through MAPK/ERK. We performed experiments using U0126, an inhibitor of MAPK kinase 1/2 (MEK1/2) [43,44]. The inhibitory effect of U0126 on the hesperetin-induced recovery of process elongation suggests that the beneficial effects of hesperetin are mediated, at least in part, through its signaling pathway (Figure 10).

Together, these results indicate that hesperetin mitigates aggregated Aβ species-induced impairment of neuronal morphological differentiation. These findings suggest that the effects of hesperetin on neuronal process elongation are associated with modulation of the MAPK/ERK signaling pathway, a cascade known to be vulnerable to Aβ-induced toxicity but targetable by flavonoid compounds [25,26].

## 3. Discussion

In the present study, we established an in vitro model of early AD-related blunted neuronal process elongation using N1E-115 cells and primary cortical neurons. Aggregated Aβ (25–35) species, as well as Aβ (1–42), species primarily mediate a mild but definite impairment of process elongation. This morphological deficit occurred in a concentration-dependent manner and was accompanied by decreased expression of neuronal differentiation markers and altered differentiation-related intracellular signaling activity. Importantly, the aggregated Aβ-induced blunted process elongation was effectively recovered by hesperetin, suggesting that some early disturbances caused by Aβ in vitro are not only potentially detectable but also pharmacologically reversible at the marker molecular and cellular levels.

A key feature of the present model is that aggregated Aβ exposure significantly impaired process elongation without inducing overt degenerative morphology or substantial cell death. This distinguishes our system from models representing advanced neurodegeneration and instead reflects cellular alterations more consistent with early-stage conditions [7,8,9,10]. In the clinical context, the brain undergoes a “synaptic failure” phase where, despite the maintenance of neuronal cell bodies, the density of neurites and synaptic connections gradually decreases [1,2]. We define our “MCI-like cellular model” based on the observation that a sub-lethal concentration of aggregated Aβ (approximately 13 µM) specifically arrests the dynamic elongation of neuronal processes while keeping the cells viable. This model effectively simulates the transitional window where structural plasticity is compromised before the onset of irreversible neurodegeneration [7,10]. In the early phases of AD, neurons remain moderately viable but exhibit reduced connectivity and gradually diminished structural plasticity. Our findings suggest that Aβ might compromise the intrinsic capacity for process extension at this stage, thereby limiting the ability of neuronal networks to compensate for emerging cellular disturbances. Such subtle morphological alterations, which are increasingly recognized as early biomarkers of cognitive decline [5,11], could reduce neuronal connectivity and weaken the resilience of neuronal processes and their networks, ultimately contributing to progressive cognitive decline. Collectively, these results support the notion that Aβ-mediated impairment may occur before overt neurodegeneration. This subtle morphological assessment was further facilitated by the CellArray-Heart culture system. The micropatterned surface aligned the cells, allowing clear image-based discrimination between cell bodies and neurite-like processes without fixation (Figure S5), which ensured efficient and robust quantification of Aβ-induced impairment and its recovery by hesperetin.

Process elongation in neuronal cells is fundamental for synaptic connectivity and circuit integrity in the CNS [14,18]. Its disruption weakens network architecture and impairs compensatory remodeling in response to physical and inflammatory stressors, or even injury, throughout the lifespan [19,22]. Aggregated Aβ is also known to disrupt neuronal differentiation from adult neural stem/progenitor cells [3,4]. In this study, in the presence of aggregated Aβ, neuronal process elongation was significantly attenuated, together with reduced expression levels of neuronal differentiation marker proteins such as GAP43 and Tau, which are essential for axonal growth and microtubule stability [15,16]. These results suggest that Aβ could interfere with the differentiation-related molecular machinery required for the maintenance and extension or re-extension of processes necessary for cell communication.

Although multiple intracellular pathways regulate neuronal cell morphogenesis, our data indicate that aggregated Aβ exposure alters at least one major signaling route involved in this process. In particular, we observed a reduction in active GTP-bound Ras and phosphorylation of its downstream MAPK/ERK, signaling molecules known to participate in neuronal differentiation [23,24]. Dysregulation of these signaling cascades has been implicated in various neurodegenerative and neurodevelopmental pathologies [20,21,23]. Rather than acting as a single dominant regulator, Ras and MAPK/ERK likely act as part of a broader network of signaling pathways including the Rho-kinase and PI3K/Akt pathways [32] that collectively support neuronal process elongation [16,17,23,24]. Therefore, the changes in the activities of Ras and MAPK/ERK reflect a disturbance of neuronal differentiation-related signaling more generally, consistent with the multifaceted impact of Aβ on neuronal cells.

Treatment with hesperetin effectively restored aggregated Aβ-induced impairments in process elongation. Hesperetin also recovered differentiation marker expression and intracellular signaling altered by Aβ, suggesting that its protective action involves normalization of mechanistic networks that support cellular morphologies. While the precise upstream target of hesperetin remains to be fully elucidated, its ability to restore the levels of GTP-bound Ras suggests a multi-targeted mechanism. Hesperetin, as well as other flavonoids, may act as a potent antioxidant to reduce reactive oxygen species that otherwise inhibit Ras activity, or it might indirectly modulate upstream receptor tyrosine kinases or G-protein coupled receptors that trigger the Ras-MAPK/ERK cascade [25,26]. By stabilizing these signaling hubs, hesperetin appears to reinforce the molecular scaffolds necessary for neurite extension.

Importantly, this action reflects a broader paradigm in which bioactive phytochemicals exert multi-target regulatory effects to combat complex neurodegenerative cascades. Recent evidence highlights that such natural compounds do not merely engage a single linear pathway but instead orchestrate an extensive crosstalk among oxidative stress mitigation, apoptosis regulation, and survival signaling networks [27,28,45,46]. For instance, the reduction in intracellular reactive oxygen species by polyphenolic structures frequently coincides with the downregulation of pro-apoptotic signaling and the simultaneous upregulation of cytoprotective pathways like Akt/mechanistic target of rapamycin (mTOR) or MAPK/ERK. It can thereby creat a unified molecular defense system against proteotoxic insults [27,28,45,46]. In this context, hesperetin and its related small molecules exhibit dual cellular biological activities. Hesperetin primarily plays a role in scavenging oxidative species but it also modulates a variety of intracellular signaling cascades including MAPK-dependent pathways [25,26]. Both oxidative species and abnormal regulation of intracellular signaling and subsequent apoptotic pathway activation are implicated in early AD-related pathology [7,8,9,10]. Our results extend these observations by demonstrating that hesperetin can reverse early molecular and morphological abnormalities caused by Aβ in a neuronal morphological differentiation model.

The ability of hesperetin to restore process elongation is likely to be particularly relevant in the context of early-stage AD, where neuronal cells are still present but their length is reduced [7,8,9,10]. Enhancing structural plasticity at this stage could help preserve or recover neuronal cell morphologies and networks [7,8,9,10,18,20]. Rather than acting as a neuroprotective agent solely by preventing cell death, hesperetin appears to promote structural recovery at the level of neuronal morphological differentiation. Hesperetin might help maintain intracellular homeostasis. Our in vitro model provides a useful platform for studying early AD-related cellular abnormalities and their reversibility. Although this system does not fully recapitulate the complexity of the in vivo brain environment, it allows precise dissection of how aggregated Aβ affects neuronal morphogenesis. It also provides a useful platform for evaluating small molecules that can restore these deficits. Coupled with advances in the scientific understanding and early diagnosis of AD [11,12], this approach may facilitate the identification of molecular targets and candidate compounds for early intervention. Such strategies may help restore neuronal structure and function rather than merely slow neurodegeneration.

## 4. Materials and Methods

### 4.1. Key Antibodies

Key materials used in this study are listed in Table 1.

### 4.2. Cell Line Culture and Differentiation

The mouse N1E-115 cell line (Japan Health Sciences Foundation, Tokyo, Japan) was cultured on cell and tissue culture dishes including Nunc dishes (Nunc brand, ThermoFisher Scientific, Waltham, MA, USA) in high-glucose Dulbecco’s modified Eagle medium (DMEM; Nacalai Tesque, Kyoto, Japan) containing 10% heat-inactivated fetal bovine serum (FBS; Gibco brand, ThermoFisher Scientific) and penicillin-streptomycin (PenStrep, Nacalai Tesque) in 5% CO_2_ at 37 °C [31,32]. Alternatively, cells were cultured on CellArray-Heart dishes (Oji Holdings Co., Tokyo, Japan), which consist of alternating 10-μm-wide conventional culture surfaces and 10-μm-wide stripes densely covered with 100 nm-wide convex structures without height differences. Cell bodies preferentially adhere to the striped regions, resulting in a highly aligned morphology that directs linear alignment and immobilization of cell bodies, thereby allowing clear discrimination between cell bodies with short neuritic protrusions and elongated neurite-like processes.

To induce differentiation, cells were cultured in DMEM supplemented with 0.5% FBS containing PenStrep in 5% CO_2_ at 37 °C for several days. Cells with processes longer than the length of two cell bodies were considered process-bearing, differentiated cells (i.e., differentiated cells) using ImageJ software (ver. Java 8, downloaded from https://imagej.nih.gov/). The length of the longest processes was also measured using NeuroJ add-in software for ImageJ.

Cell morphologies were captured using a smartphone-based i-NTER LENS system (Micronet Inc., Saitama, Japan) and i-NTER SHOT software (ver. 2, Micronet Inc.).

### 4.3. Primary Cell Culture and Process Elongation

Animals were handled in accordance with the ARRIVE guidelines (https://arriveguidelines.org/), and all studies were conducted according to committee-approved protocols as described in the Ethics statement section. Mice were euthanized by chemical anesthesia using an intraperitoneal injection of a mixture of 0.3 mg/kg medetomidine, 4 mg/kg midazolam, and 5 mg/kg butorphanol.

Primary cortical neuronal cells were isolated from the cerebrum of C57BL/6JJcl mice (Clea Japan, Inc., Tokyo, Japan) at embryonic days 16 to 17 and cultured as previously described [33,34]. Following incubation with 100 units/mL papain (Worthington Biochemical, Lakewood, NJ, USA) in Leibovitz’s L-15 medium (ThermoFisher Scientific) at 37 °C for 15 min, cells were gently dissociated by pipetting the medium up and down. The dissociated cells were plated at 3 to 5 × 10^5^/cm^2^ on polylysine-coated cell culture dishes (Nacalai Tesque). The culture medium consisted of Neurobasal medium supplemented with 2% B27 (Thermo Fisher Scientific), 1% GlutaMAX (Thermo Fisher Scientific), and 0.1 mg/mL gentamicin solution (Thermo Fisher Scientific). Cells were maintained in 5% CO_2_ at 37 °C. The medium was replaced with fresh medium 1 day after isolation; thereafter, half of the medium was replaced every 2 to 3 days. After maintaining the neurons for 7 to 14 days, cultured cortical neuronal cells were detached using a 0.05% trypsin solution containing 0.53 mM EDTA (ThermoFisher Scientific) and stored in liquid nitrogen until use.

To initiate experiments for observing process elongation, freshly thawed cells were plated onto cell culture dishes and allowed to elongate processes over several days. In standard experiments, on day 3 after cell seeding, cells bearing a process longer than the length of 2 cell bodies were typically classified as axon-bearing cells (i.e., differentiated cells) using ImageJ software.

### 4.4. Aggregated Aβ (25–35)-Forming Condition

Aβ (25–35) or full-length Aβ (1–42) oligomers, which represents biologically active toxic fragment of the full-length amyloid beta protein, were prepared by diluting 5 mM Aβ (amino acids 25–35 or 1–42, Peptide Institute, Inc., Osaka, Japan) in dimethyl sulfoxide to 0.1 mM in 10 mM hydrochloric acid, immediately vertexing for 30 s, and incubating at 37 °C for 24 h [37,38]. Soluble Aβ was simply dissolved in dimethyl sulfoxide and directly applied on cultured cells without additional aggregation procedures.

### 4.5. Polyacrylamide Gel Electrophoresis and Immunoblotting

Cells were lysed in a buffer containing 50 mM HEPES-NaOH, pH 7.5, 150 mM NaCl, 5 mM MgCl_2_, 1 mM dithiothreitol (DTT), 1 mM phenylmethylsulfonyl fluoride (PMSF), 0.02 mM leupeptin, 1 mM EDTA, 1 mM Na_3_VO_4_, 10 mM NaF, and 0.5% NP-40. For denaturation, centrifugally collected cell supernatants from each sample were denatured in premade sample buffers (Fujifilm Wako Chemicals, Tokyo, Japan) [35,36]. Samples were then separated using premade gels for SDS-PAGE (Nacalai Tesque).

Electrophoretically separated proteins were transferred to a polyvinylidene difluoride (PVDF) membrane (Millipore, Rahway, NJ, USA), blocked with Blocking One (Nacalai Tesque), and immunoblotted using primary antibodies, followed by peroxidase enzyme-conjugated secondary antibodies. Peroxidase-reactive bands were captured and scanned using a CanoScan LiDE 400 system (Canon, Tokyo, Japan) with ScanGear software (ver. MacOS14, Canon). For immunoblotting analysis, we performed several sets of experiments. Immunoreactive bands were quantified using ImageJ software, normalizing signal intensities of the targeted bands to those of other control immunoreactive bands. The representative blots shown in the figures are from several blots. Full size blots are shown in Figures S6–S14.

### 4.6. Pull-Down Assay for GTP-Bound Ras

Cells were lysed in a buffer containing 50 mM HEPES-NaOH, pH 7.5, 150 mM NaCl, 10 mM MgCl_2_, 1 mM DTT, 1 mM PMSF, 0.02 mM leupeptin, 1 mM EDTA, 1 mM Na_3_VO_4_, 10 mM NaF, and 0.5% NP-40. Active, GTP-bound Ras, which was pulled down using a recombinant Ras-binding domain (RBD) of Raf-1 [41,42,43,44], was applied to SDS-PAGE gels, transferred to a PVDF membrane, and immunoblotted with an anti-pan-Ras antibody. Ras levels in the lysates were also used for immunoblotting with an anti-pan-Ras antibody.

### 4.7. Statistical Analysis

Values are presented as means ± standard deviation (SD) from independent experiments. Intergroup comparisons were performed using the unpaired Student’s *t*-test in Excel software (ver. 2023, Microsoft, Redmond, WA, USA). One-way analysis of variance was followed by Tukey’s honestly significant difference test, using a StatPlus add-in software for Excel (ver. 2021, Alexandria, VA, USA) when multiple comparisons were necessary. Differences were considered statistically significant when *p* < 0.05.

### 4.8. Ethics Statement

Techniques using genetically modified cells and related procedures were performed in accordance with protocols approved by the Tokyo University of Pharmacy and Life Sciences Gene and Animal Care Committee (Nos. LS28-20 and LSR3-011, approved on 1 April 2025).

## 5. Conclusions

Our study demonstrates that aggregated Aβ induces early, reversible impairments in neuronal process elongation and differentiation-related signaling. Hesperetin effectively counteracts these abnormalities, highlighting its potential as a modulator of early AD-related cellular dysfunction. These findings emphasize the importance of targeting early, pre-degenerative stages of this disease and support the use of an in vitro differentiation model as a possible platform for discovering compounds and molecular pathways involved in the recovery of cell structure and function.

### Limitations and Future Directions

Despite the strengths of the present in vitro model, several limitations should be acknowledged.

First, although the N1E-115 cell line is a valuable tool for characterizing neurite outgrowth, our experiments were mostly limited to this in vitro system. As such, it may not reflect the full complexity of the in vivo brain environment, where various cell types and extracellular factors interact. This system lacks the interactions with glial cells, vascular components, and immune signaling that are known to influence AD pathology. Importantly, it does not account for the complex inflammatory dysregulation primarily observed in early AD. Future studies utilizing advanced co-culture systems (e.g., neuronal cells with astrocytes or microglia) will be essential to address these multicellular interactions and neuroinflammatory mechanisms. Therefore, caution should be exercised when extrapolating these results to whole-organism physiology.

Second, we did not use transgenic or Aβ-based mouse models of AD in this study. Therefore, the in vivo relevance of the observed protective effects of hesperetin on neuronal morphology and signaling remains to be validated in whole-animal systems where network-level dysfunction, inflammation, and behavioral outcomes can be assessed.

Furthermore, the present study did not incorporate human-derived neuronal models, including induced pluripotent stem cell (iPSC)-derived neurons. Future studies leveraging patient-specific iPSC-derived neuronal and glial cells will be critical to determining whether the Aβ-induced impairments observed in our model and their reversal by hesperetin are reproducible in a human cellular context. Such systems would also facilitate examination of disease-relevant genetic backgrounds and inter-individual variability, thereby enabling a more rigorous and translational evaluation of the therapeutic potential of hesperetin and related compounds.

## Figures and Tables

**Table 1:** Key materials used in this study.

Reagents or Materials	Companies or Sources	Cat. Nos.	Lot. Nos.	Concentrations Used
Key antibodies				
Ant-growth-associated protein 43 (GAP43)	Santa Cruz Biotechnology (Santa Cruz, CA, USA)	sc-17790	J0920	Immunoblotting (IB), 1:5000
Anti-Tau	Santa Cruz Biotechnology	sc-21796	J2524	IB, 1/500
Anti-glyceraldehyde-3-phosphate dehydrogenase (GAPDH)	Santa Cruz Biotechnology	sc-32233	J2523	IB, 1:5000
Anti-phospho-p44/42 MAPK (Erk1/2) (Thr202/Tyr204) antibody	Cell Signaling Technology (Danvers, MA, USA)	4370	30	IB, 1:2000
Anti-p44/42 MAPK (Erk1/2) antibody	Cell Signaling Technology	4695	35	IB, 1:1000
Anti-pan Ras (N-Ras, H-Ras, and K-Ras)	Santa Cruz Biotechnology	sc-16691	A0824	IB, 1:250
Anti-synaptophysin	Proteintech Group (Rosemont, IL, USA)	17785-1-AP	00183734	IB, 1:250
Anti-postsynaptic density protein 95 (PSD95)	Proteintech Group	20665-1-AP	00183521	IB, 1:250
Anti-heme oxygenase 1 (HO-1)	Proteintech Group	1-701-1-AP	00180850	IB, 1:250
Anti-4-hydroxy-2-nonenal (4-HNE)	JalCA (Shizuoka, Japan)	MHN-020P	014	IB, 1:10
Anti-cytochrome c	Santa Cruz Biotechnology	sc-13560	K1022	IB, 1:1000
Anti-rabbit IgG (goat) pre-absorbed peroxidase-conjugate	Nacalai Tesque (Kyoto, Japan)	21858-24	M3H6046	IB, 1:20,000
Anti-mouse IgG (goat) pre-absorbed peroxidase-conjugate	Nacalai Tesque	21860-61	L4B5968	IB, 1:20,000
Key materials				
Amyloid β protein amino acids 25-35 (human)	Peptide Institute (Osaka, Japan)	4309	710410	13 μM
Amyloid β protein amino acids 1-42 (human)	Merck Millipore (Darmstadt, Germany)	PP69-.25MG	4328565	13 μM
Nacalai Tesque Trypan Blue Stain Solution (0.5%)	Nacalai Tesque	29853-34	L4R2629	0.005%
Recombinant DNA				
pEGFP-C1 (mamalian cell GFP expresssion plasmid)	Takara Bio (Kyoto, Japan; discontinued product)	N/A	N/A	0.75 mg of DNA per 6 cm dish

## Data Availability

The datasets used and/or analyzed for the current study are available from the corresponding author upon reasonable request as well as the Figshare website, https://doi.org/10.6084/m9.figshare.31857220.

## Supplementary Materials

The following supporting information can be downloaded at: https://www.mdpi.com/article/10.3390/ijms27125481/s1.
