# Supplementary material for: Hesperetin Rescues Amyloid Beta-Induced Defects in Neurite Outgrowth Under In Vitro Mild Cognitive Impairment-like Cellular Conditions
Source: Int J Mol Sci. 2026 Jun 17;27(12):5481. doi: 10.3390/ijms27125481 (PMC13299210; doi:10.3390/ijms27125481)
Supplement: Supplementary file 1 [file ijms-27-05481-s001.zip › supplemental figure legends.pdf]

Supplemental figure legends

Figure S1. Addition of aggregated Aβ (25–35) inhibits morphological differentiation in N1E-115 cells in a concentration-dependent manner. (A) N1E-115 cells were treated with the indicated concentrations of aggregated Aβ (25–35). Cells were allowed to differentiate morphologically for 3 days (** $p < 0.01$; n = 10 fields). In the upper eight images, typically differentiated or undifferentiated cells are surrounded by dotted red lines. In the lower eight images, cell bodies are surrounded by dotted white lines. Dying cells incorporated trypan blue dye (blue). (B) The percentages of trypan blue dye–positive cells increased in an Aβ concentration-dependent manner (blue in graph). Cells with processes were counted as differentiated and statistically depicted in the graph (red) (** $p < 0.01$; n = 10 fields).

Figure S2. Treatment with aggregated Aβ (25–35) affects the levels both of expression of synaptophysin, PSD95, and HO-1 and of 4-HNE modification. (A, B) N1E-115 cells were treated with (+) 13 μM aggregated Aβ (25–35) or its vehicle control (-), in the presence (+) of 10 μM hesperetin or its vehicle control (DMSO). Following the induction of differentiation, cells were collected at day 3 and immunoblotted with antibodies against synaptophysin, PSD95, HO-1, 4-HNE modified protein, and an internal control protein (GAPDH). The quantified immunoreactive bands were normalized to internal control proteins (** $p < 0.01$; n = 3).

Figure S3. Treatment with aggregated Aβ (1–42) inhibits the expression levels of differentiation marker proteins. (A, B) N1E-115 cells were treated with (+) 13 μM aggregated Aβ (1–42) or its vehicle control (-), in the presence (+) of 10 μM hesperetin or its vehicle control (DMSO). Following the induction of differentiation, cells were collected at day 3 and immunoblotted with antibodies against synaptophysin, PSD95, and an internal control protein (GAPDH). Quantified immunoreactive bands were normalized to internal control proteins (** $p < 0.01$; n = 3).

Figure S4. Hesperetin concentration-dependently restores the expression of differentiation marker proteins decreased by aggregated Aβ (25–35). (A, B) N1E-115 cells were treated with (+) aggregated Aβ (25–35) in the presence of 0 (DMSO), 3, 10, 30, or 100 μM hesperetin. Following the induction of differentiation, cells were collected at day 3 and immunoblotted with antibodies against synaptophysin, PSD95, cytochrome c, and an internal control protein (GAPDH). The quantified immunoreactive bands were normalized to internal control proteins (** $p < 0.01$; n = 3).

Figure S5. N1E-115 cells exhibit an neurite extension on CellArray-Heart dishes. (A, B) N1E-115 cells were placed on CellArray-Heart dishes and treated with (+) 13 μM aggregated Aβ (25–35) or its vehicle control (-), in the presence (+) of 10 μM hesperetin or its vehicle control (DMSO). Cells were allowed to differentiate morphologically for 0 or 3 days. The longest processes were measured and depicted in the graph. (** $p < 0.01$; n = 100 cells randomly selected from three independent cell culture dishes).

Figure S6. Computer-saved images of Figure 1.

Figure S7. Computer-saved images of Figure 2.

Figure S8. Computer-saved images of Figure 3.

Figure S9. Computer-saved images of Figure 6.

Figure S10. Computer-saved images of Figure 7.

Figure S11. Computer-saved images of Figure 9.

Figure S12. Computer-saved images of Figure S2.

Figure S13. Computer-saved images of Figure S3.

Figure S14. Computer-saved images of Figure S4.
