# Supplementary figures and images for: Hesperetin Rescues Amyloid Beta-Induced Defects in Neurite Outgrowth Under In Vitro Mild Cognitive Impairment-like Cellular Conditions
Source: Int J Mol Sci. 2026 Jun 17;27(12):5481. doi: 10.3390/ijms27125481 (PMC13299210; doi:10.3390/ijms27125481)

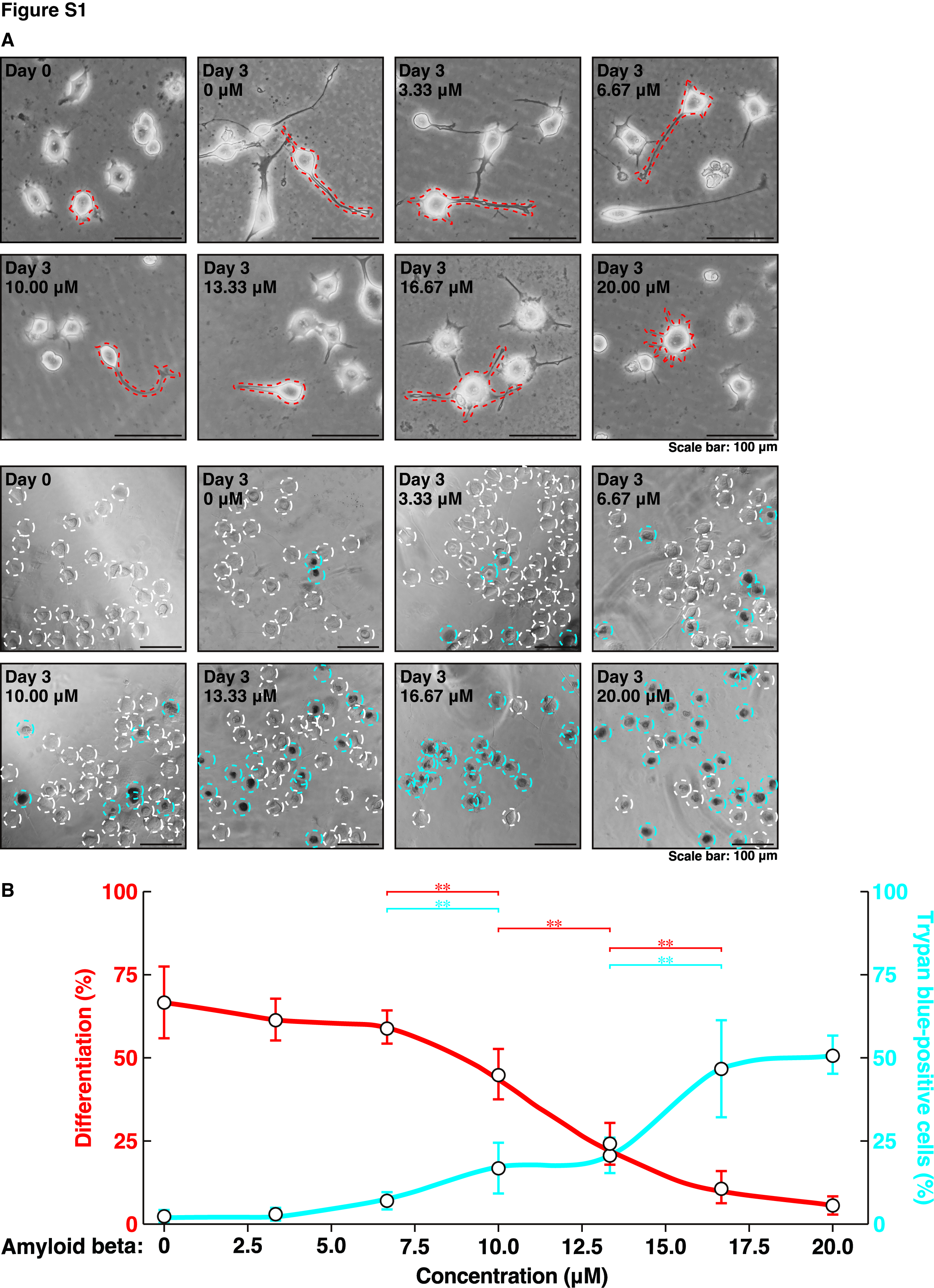

Supplement: Supplementary file 1 [file ijms-27-05481-s001.zip › Figure S1.tif]

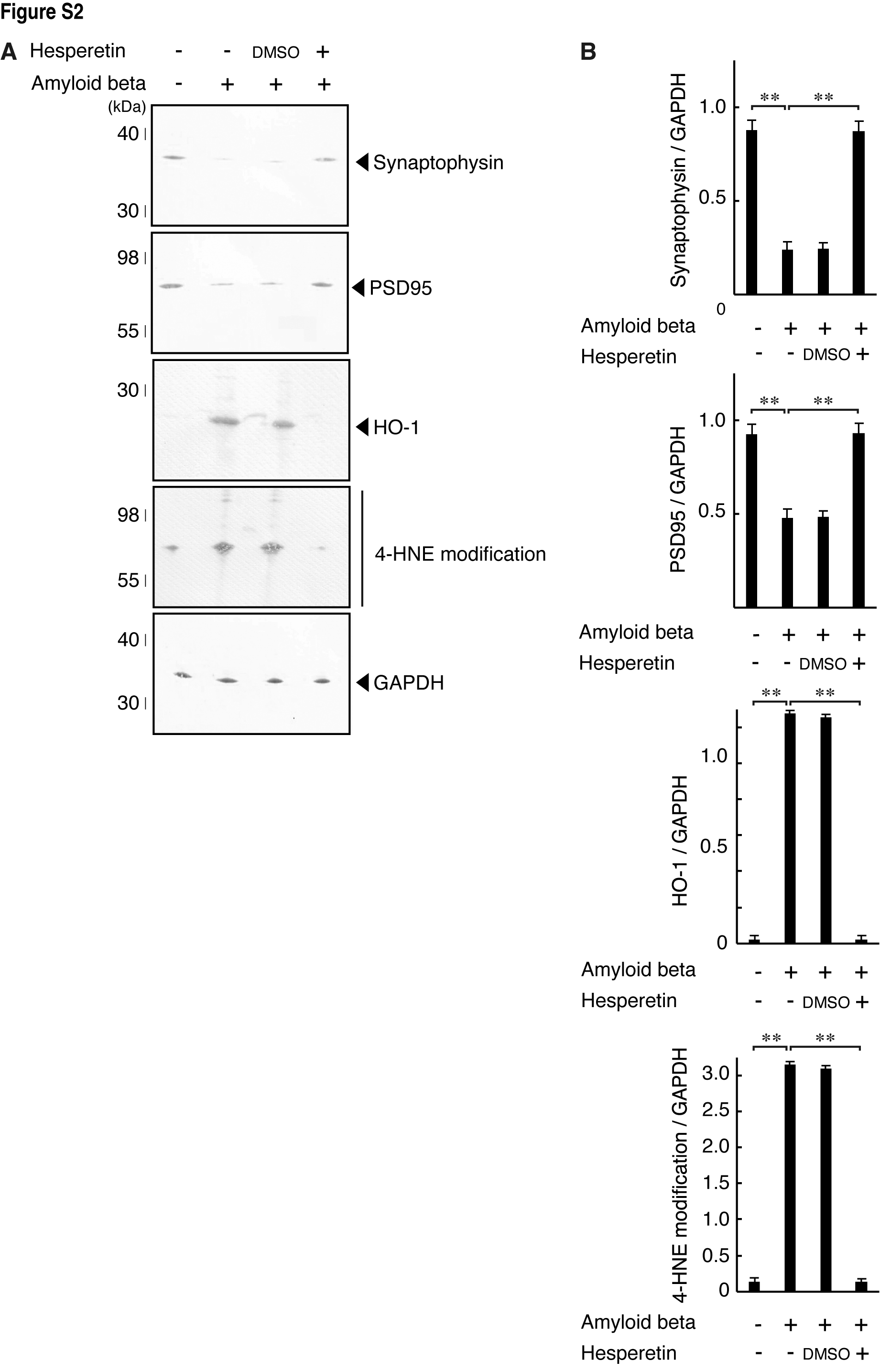

Supplement: Supplementary file 1 [file ijms-27-05481-s001.zip › Figure S2.tif]

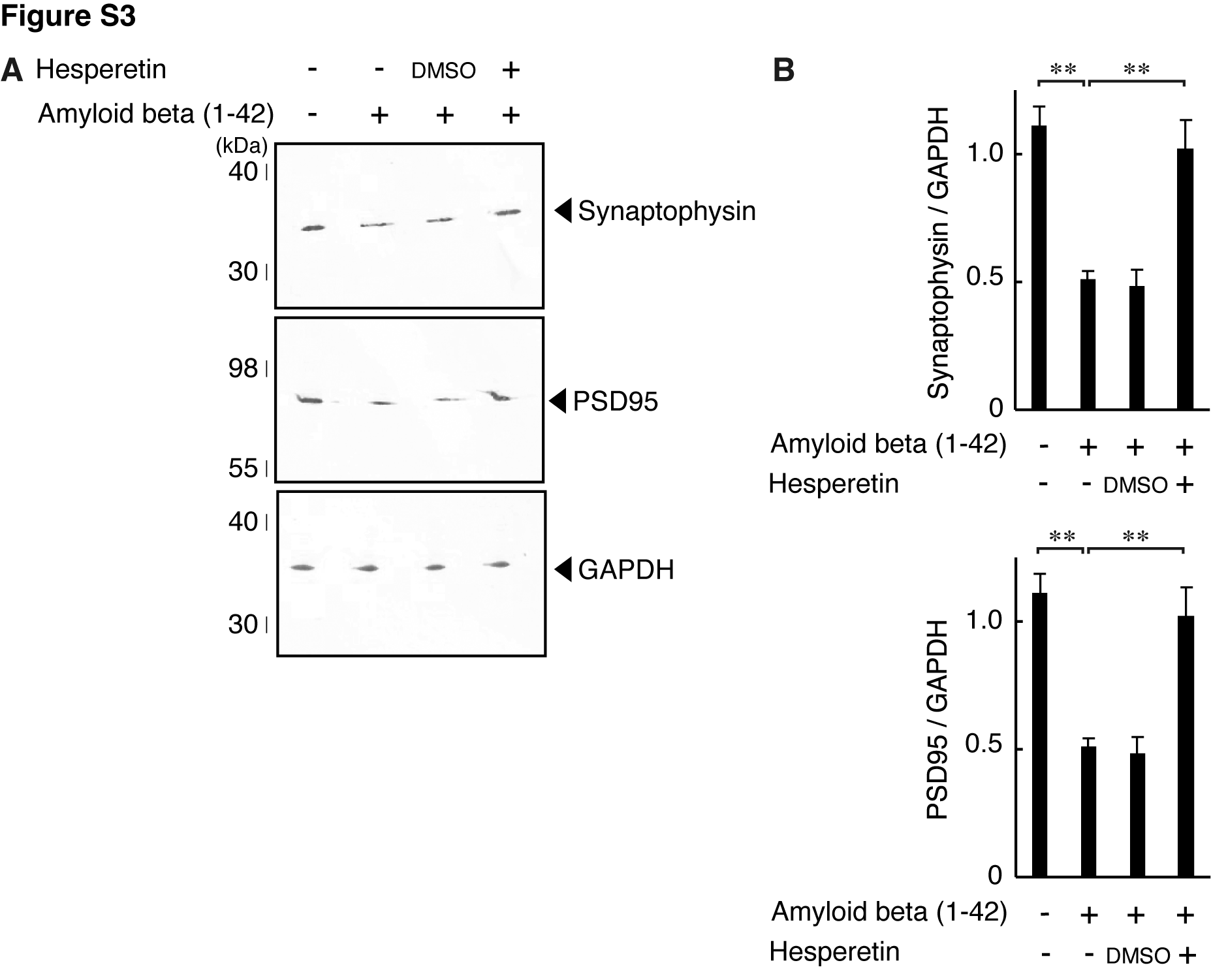

Supplement: Supplementary file 1 [file ijms-27-05481-s001.zip › Figure S3.tif]

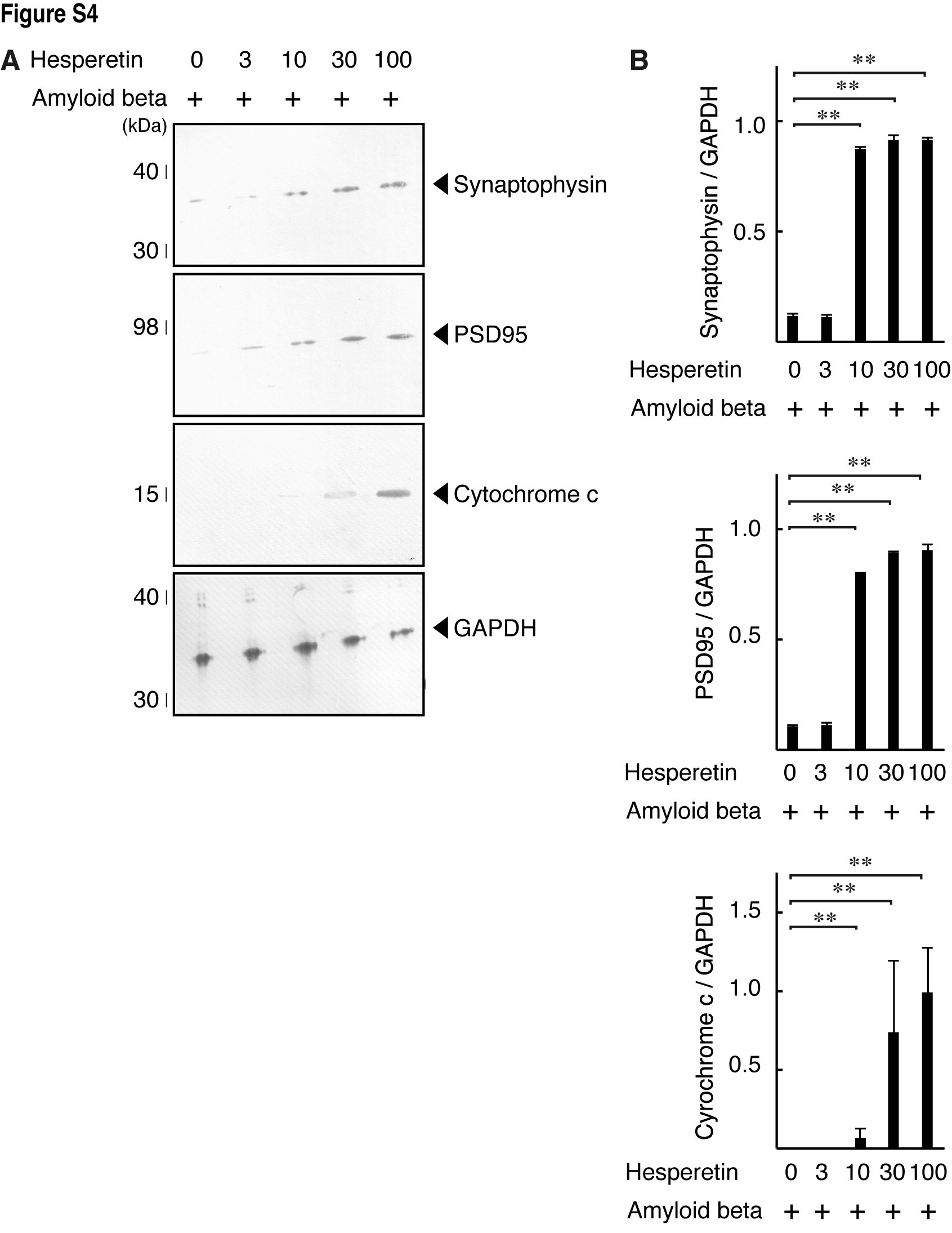

Supplement: Supplementary file 1 [file ijms-27-05481-s001.zip › Figure S4.tif]

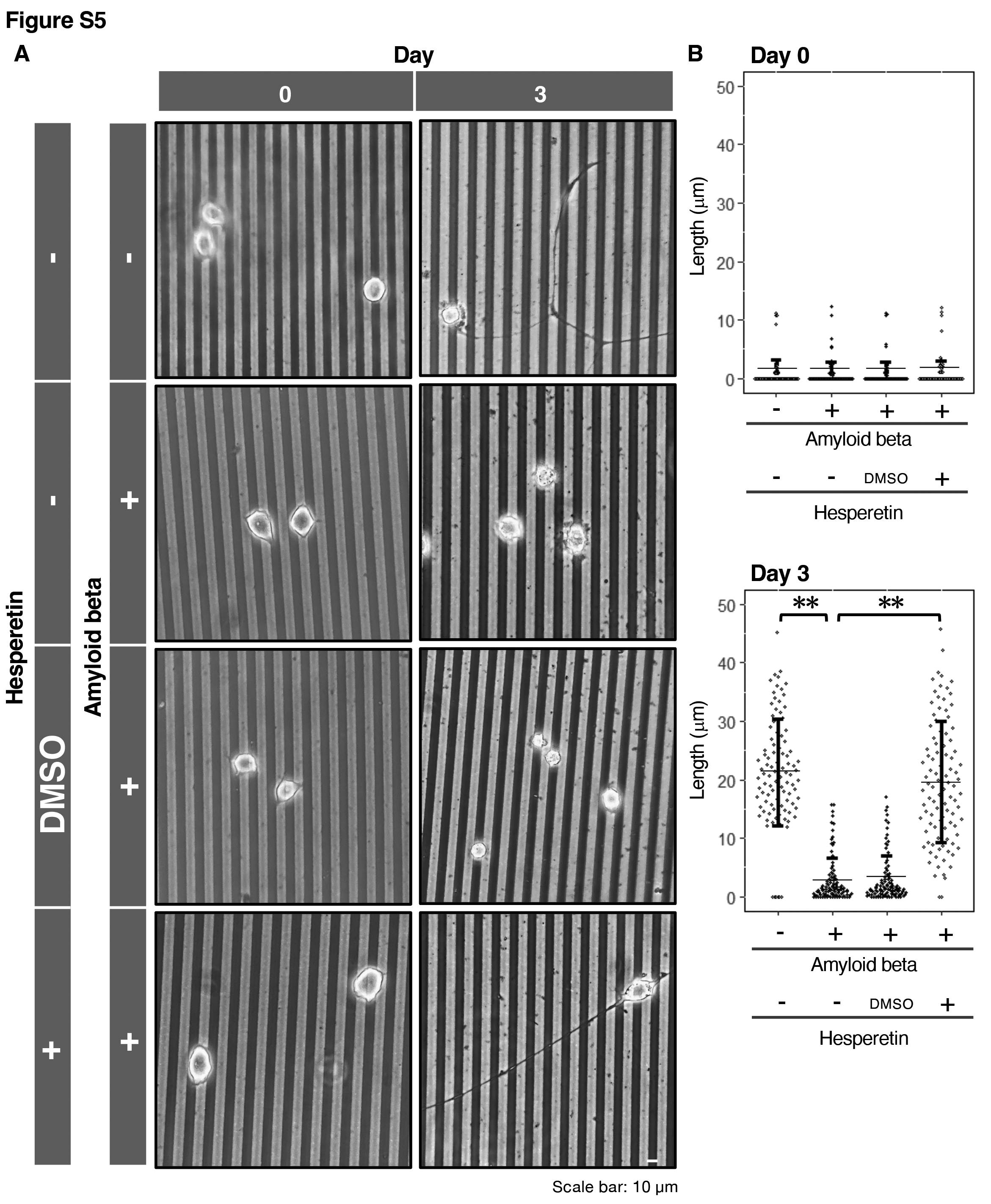

Supplement: Supplementary file 1 [file ijms-27-05481-s001.zip › Figure S5.tif]

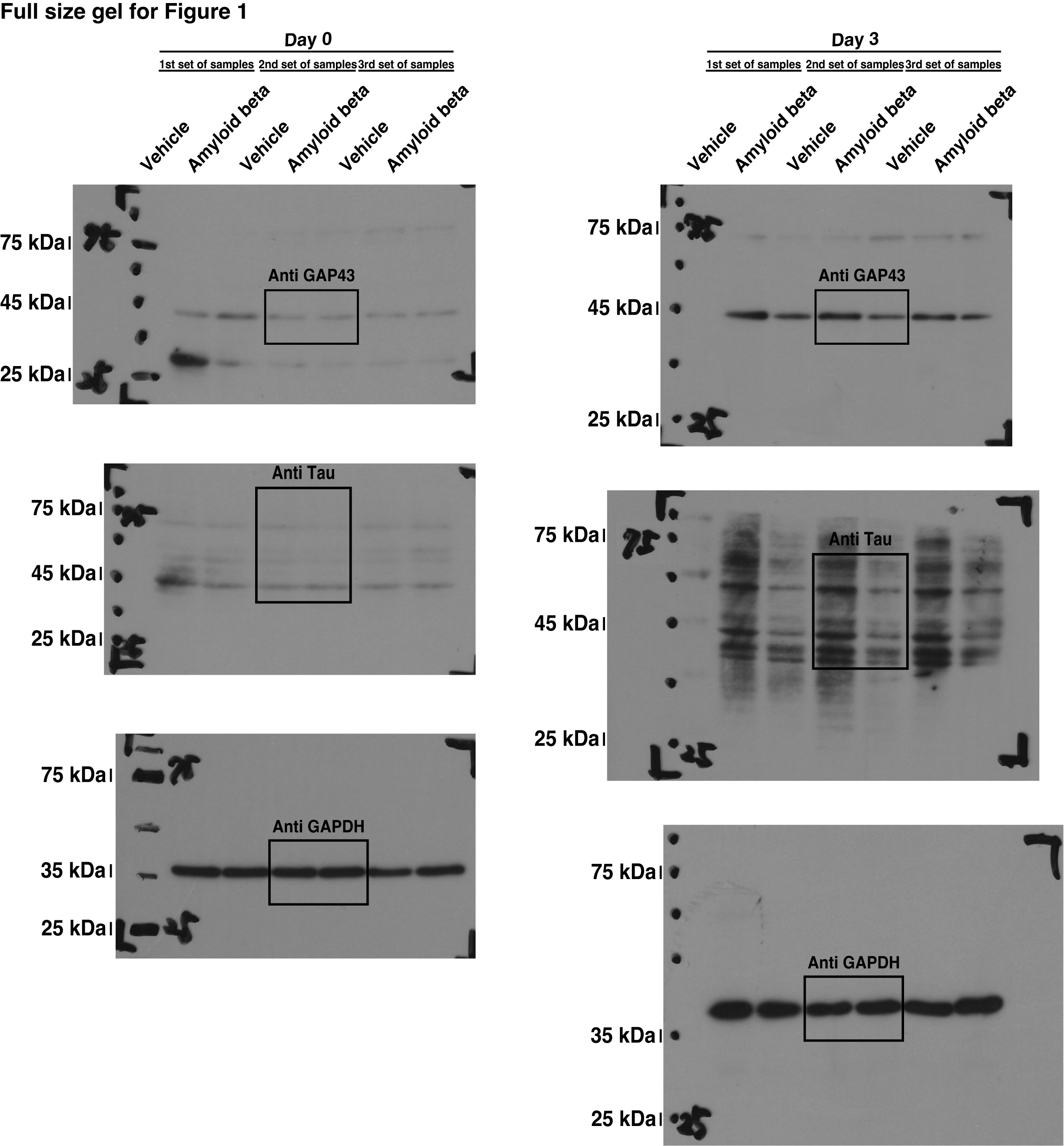

Supplement: Supplementary file 1 [file ijms-27-05481-s001.zip › Figure S6 Full size gel for Figure 1.tif]

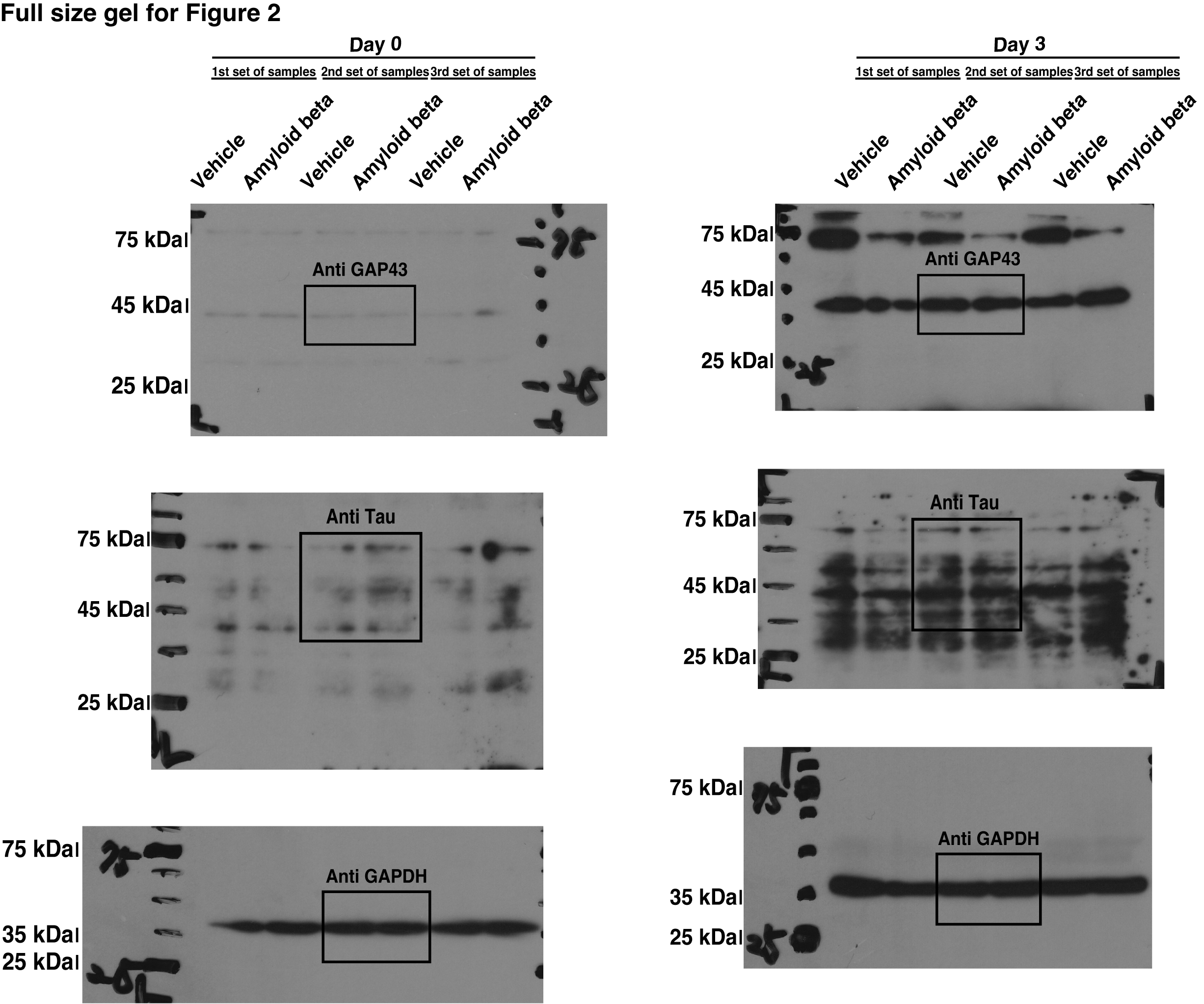

Supplement: Supplementary file 1 [file ijms-27-05481-s001.zip › Figure S7 Full size gel for Figure 2.tif]

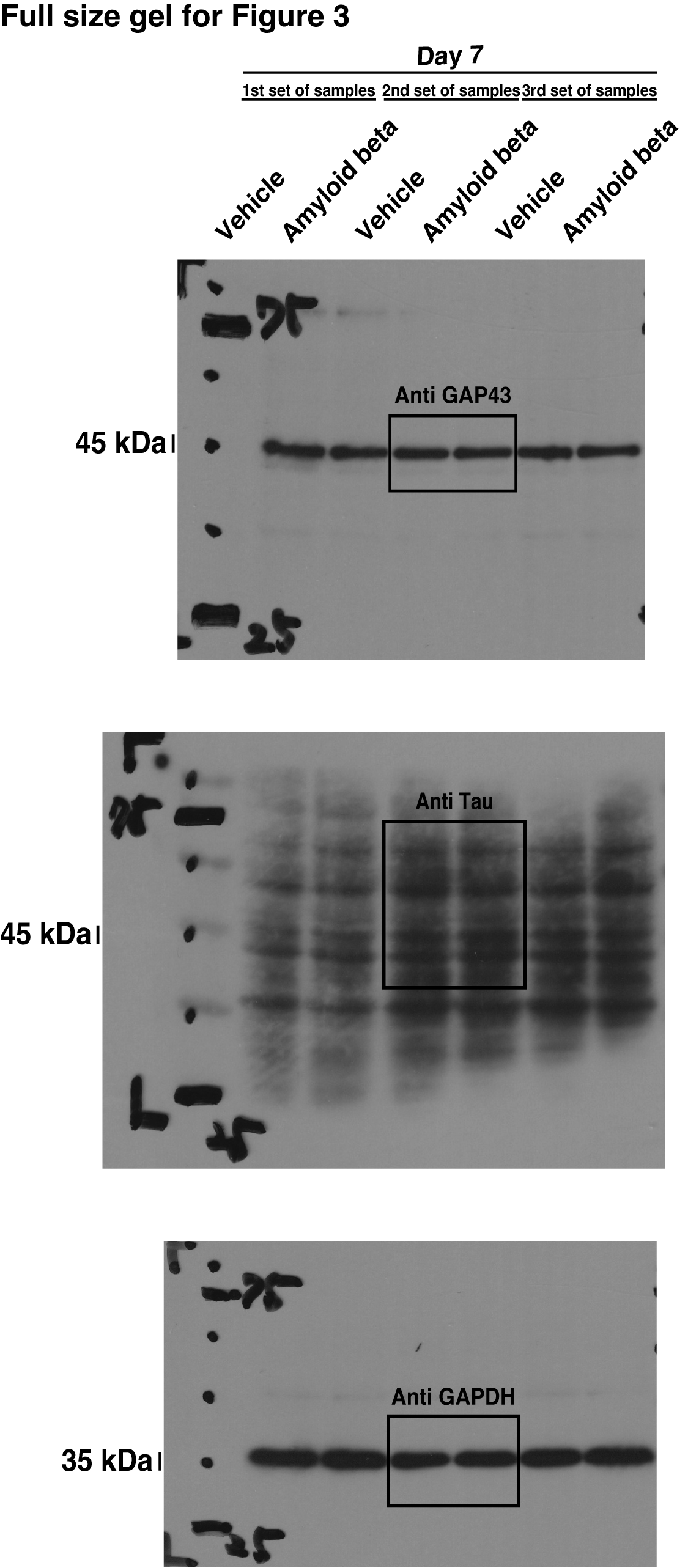

Supplement: Supplementary file 1 [file ijms-27-05481-s001.zip › Figure S8 Full size gel for Figure 3.tif]

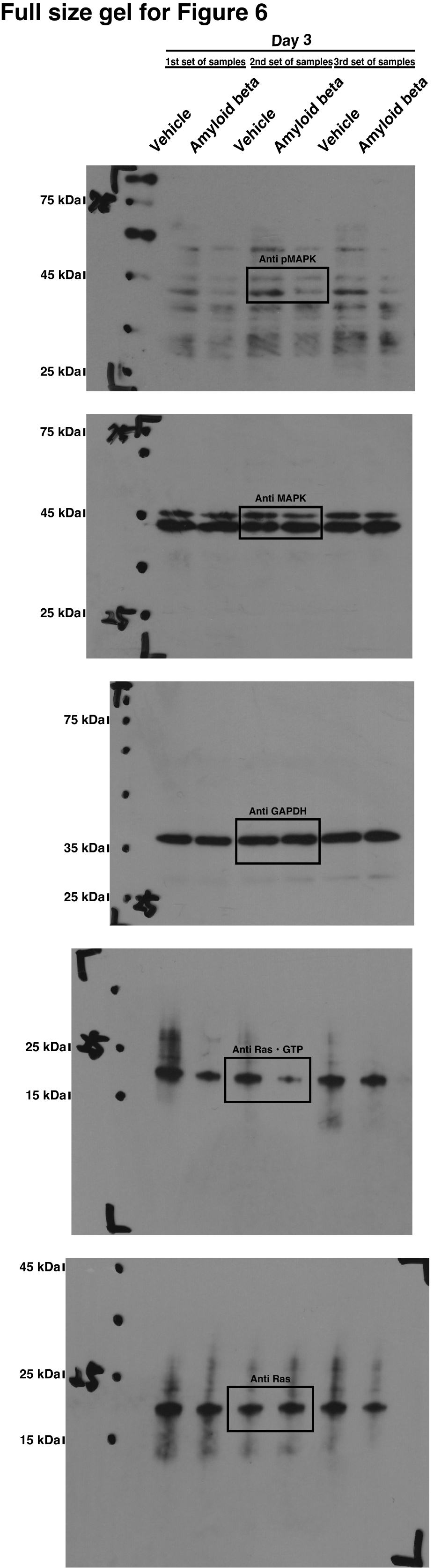

Supplement: Supplementary file 1 [file ijms-27-05481-s001.zip › Figure S9 Full size gel for Figure 6.tif]

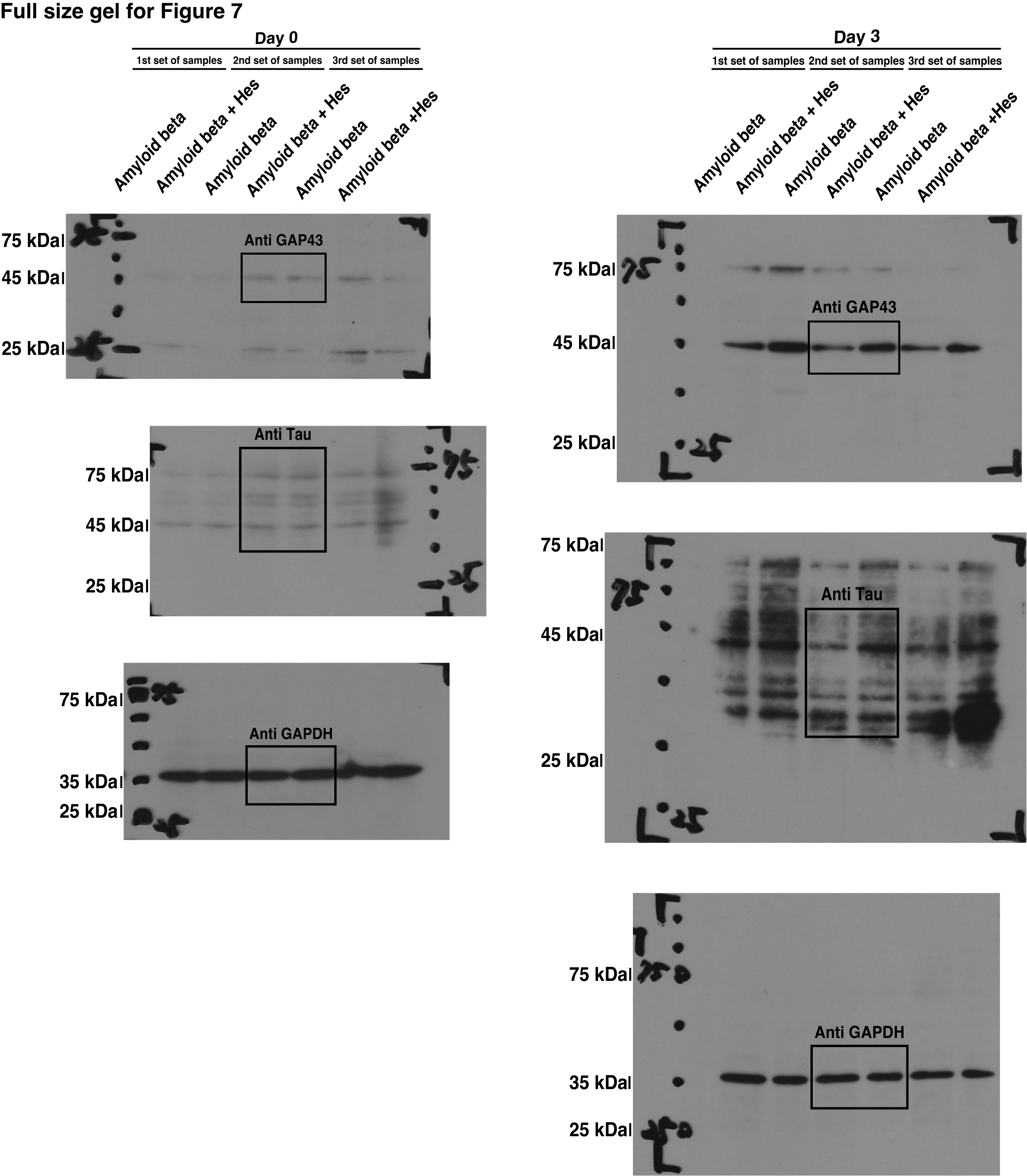

Supplement: Supplementary file 1 [file ijms-27-05481-s001.zip › Figure S10 Full size gel for Figure 7.tif]

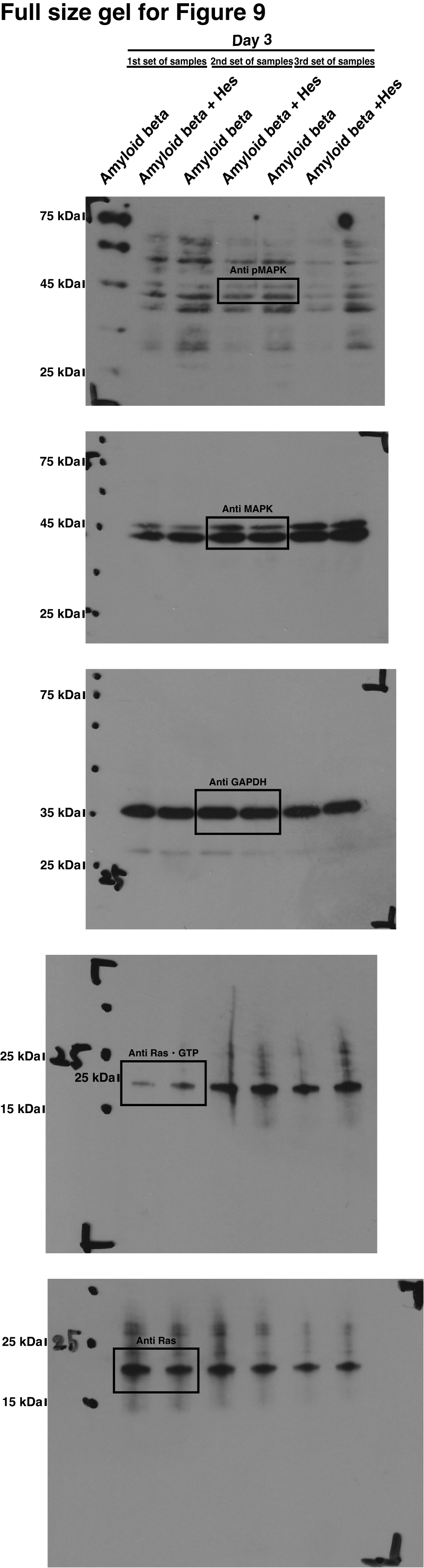

Supplement: Supplementary file 1 [file ijms-27-05481-s001.zip › Figure S11 Full size gel for Figure 9.tif]

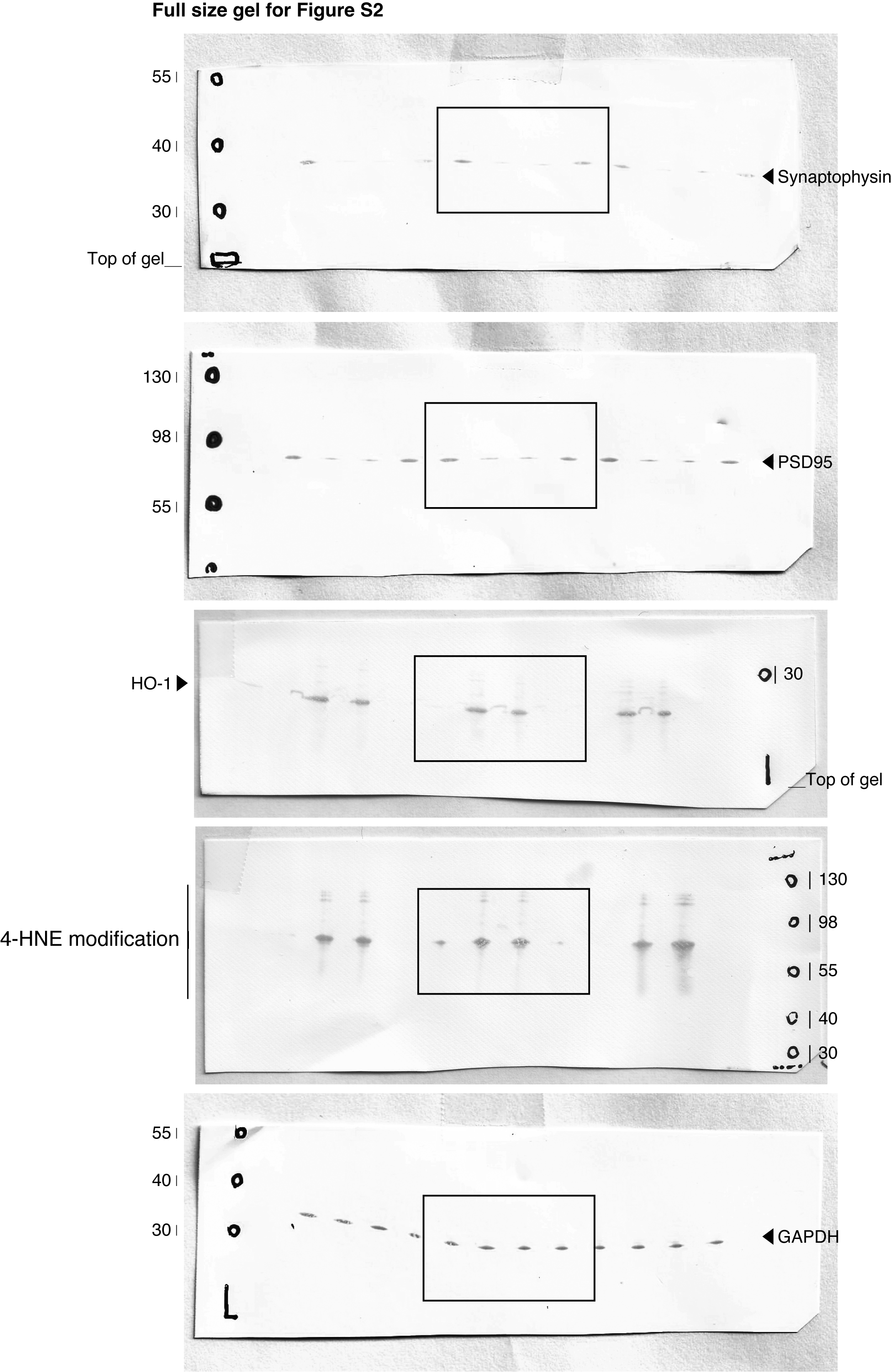

Supplement: Supplementary file 1 [file ijms-27-05481-s001.zip › Figure S12 Full size gel for Figure S2.tif]

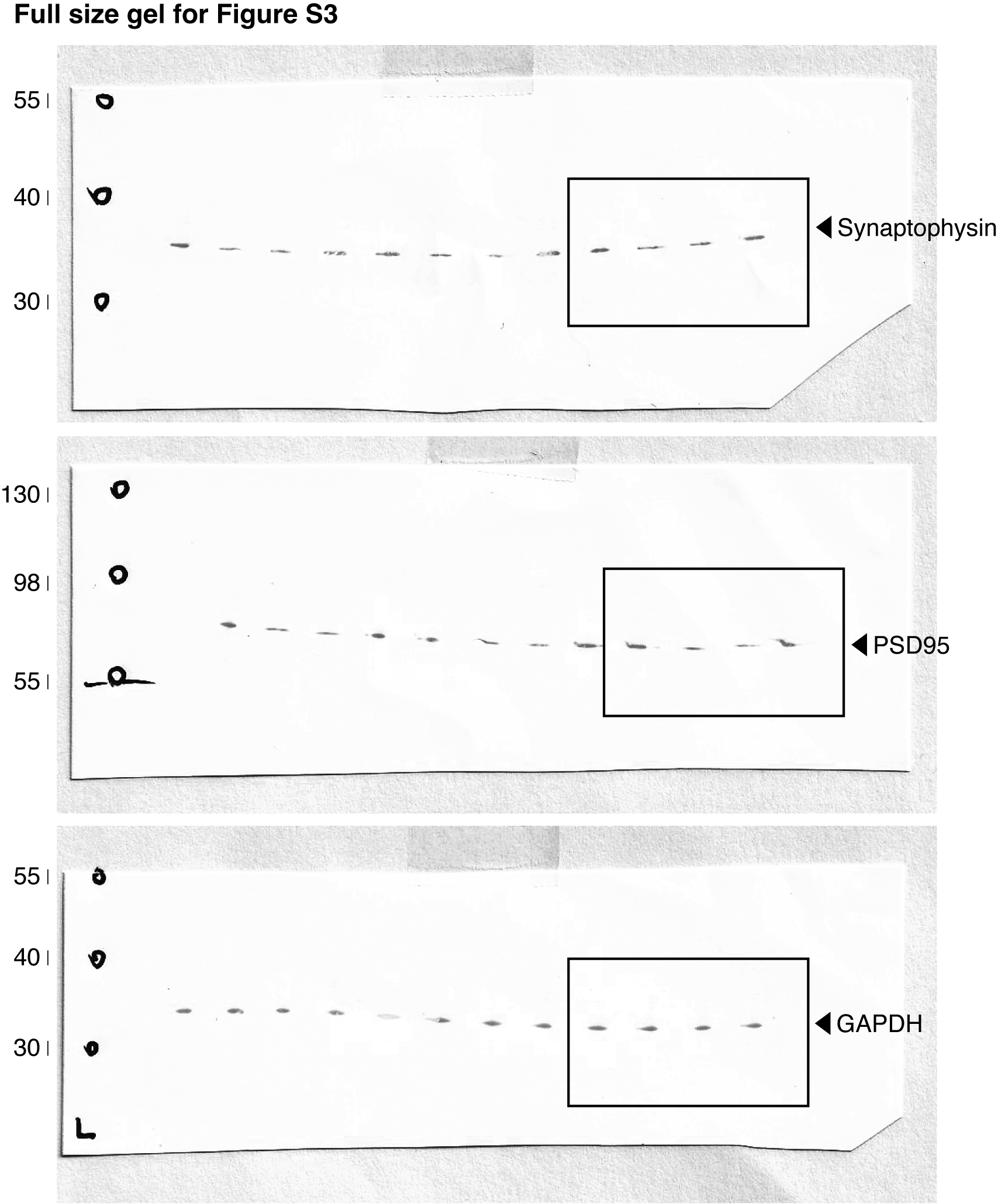

Supplement: Supplementary file 1 [file ijms-27-05481-s001.zip › Figure S13 Full size gel for Figure S3.tif]

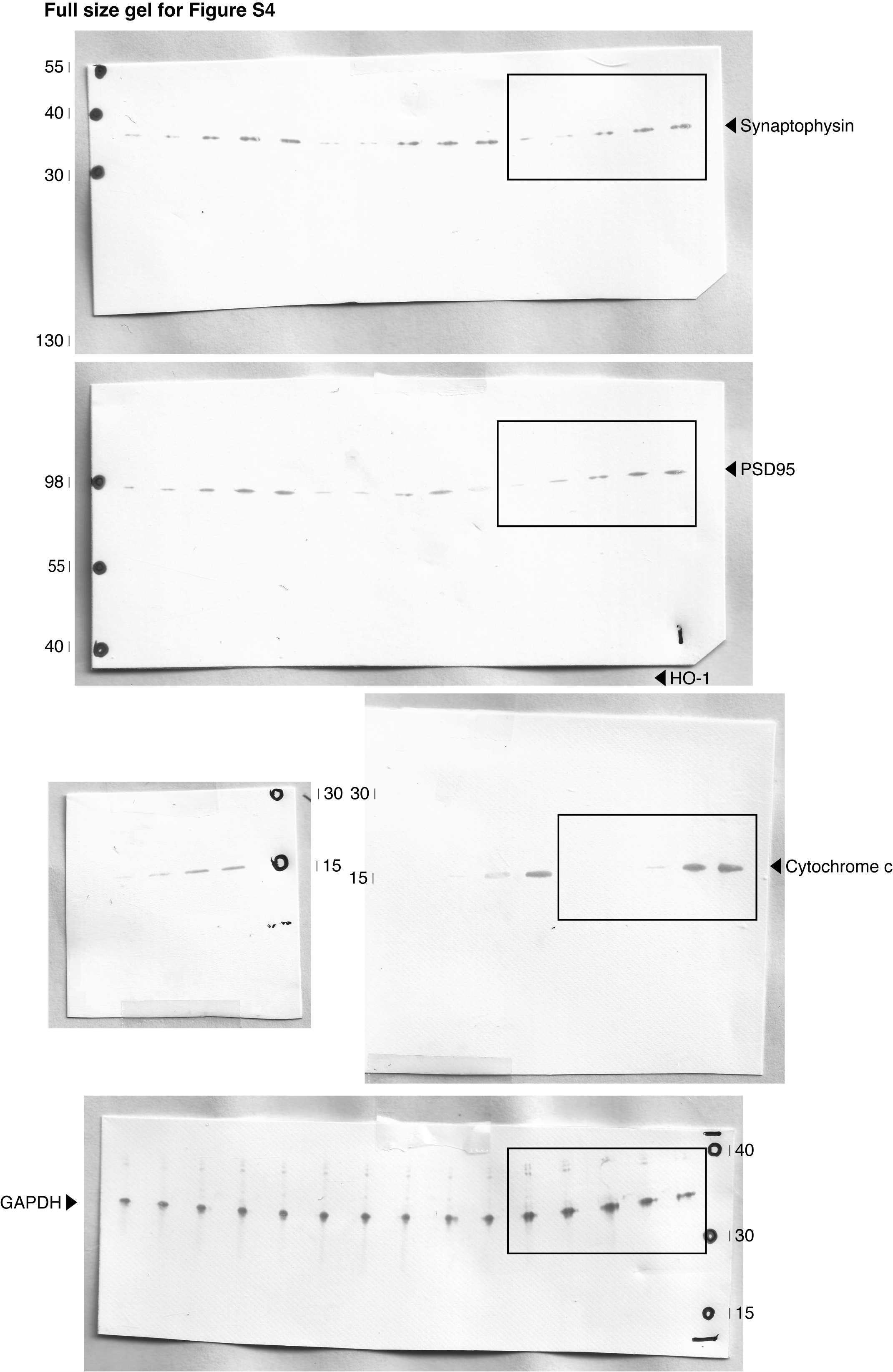

Supplement: Supplementary file 1 [file ijms-27-05481-s001.zip › Figure S14 Full size gel for Figure S4.tif]
